# AI-driven cybersecurity for industrial internet of things: architectures, challenges, datasets, and future research directions

**DOI:** 10.3389/fdata.2026.1938279

**Published:** 2026-09-07

**Authors:** Siddhartha Singhal, Kakelli Anil Kumar

**Affiliations:** School of Computer Science and Engineering, Vellore Institute of Technology, Vellore, Tamil Nadu, India

**Keywords:** cybersecurity, deep learning, edge intelligence, explainable artificial intelligence, federated learning, graph neural networks, Industrial Internet of Things (IIoT), intrusion detection systems

## Abstract

While the Industrial Internet of Things (IIoT) has a wide range of applications in the modern era, including smart manufacturing, healthcare, transportation, energy, and critical infrastructure, the multitude of devices and distributed communication, alongside the convergence of cyber and physical systems, makes these environments vulnerable to more advanced cyber-attacks. Traditional signature or pattern-based security solutions continue to be ineffective against new, sneaky, and zero-day attack strategies, fueling the interest in AI-powered cybersecurity. This review aims to analyze the latest developments systematically in intelligent threat detection and defense in IIoT environments. The review critically analyzes the cybersecurity research published over the past few years (2020–2026) on cyber threats across the various layers of the IIoT architecture, publicly available cybersecurity datasets, evaluation practices, and AI-based intrusion detection methods, such as machine learning, deep learning, hybrid architectures, transformers, graph neural networks, federated learning, reinforcement learning, and explainable AI. High benchmark performance alone is not sufficient to claim cybersecurity effectiveness, as the synthesis shows persistent limitations in cross-domain generalization, computational overhead, explainability, adversarial robustness, edge deployment, and operational validation. Emerging research priorities included in this review are lightweight edge intelligence, continual and adaptive learning, explainable federated intelligence, digital-twin-enabled security, foundation-model-driven cyber intelligence, autonomous cyber defense, and trustworthy AI. This review provides a pathway toward resilient, adaptive, and operationally deployable cybersecurity solutions for next-generation IIoT and highlights the PRISMA-based identification process.

## 1 Introduction

The rapid evolution of the IIoT is transforming modern industrial ecosystems by enabling intelligent automation, continuous monitoring, predictive maintenance, automated decision-making, and large-scale interconnected cyber–physical infrastructures. Industrial sectors such as smart manufacturing, healthcare, transportation, energy systems, logistics, oil and gas, and critical infrastructure rely on IIoT technologies to deliver better operational efficiency, scalability, reliability, and data-driven industrial intelligence. The amalgamation of computing at the edge, cloud platforms, software-defined networks, cyber–physical systems, and artificial intelligence has further accelerated the evolution of highly connected industrial environments that enable autonomous operation and intelligent orchestration of industrial processes (Ba, 2025; Liu et al., 2026; Holdbrook et al., 2024).

Notwithstanding these technological breakthroughs, an extensive implementation of IIoT systems has also broadened the cybersecurity attack surface of industrial infrastructure. IIoT ecosystems differ from traditional enterprise networks, as they are composed of highly heterogeneous, distributed, resource-constrained devices that are always interconnected and communicate via a variety of industrial protocols and network topologies. These environments produce vast amounts of heterogeneous data streams that must meet very stringent latency, reliability, and availability constraints. As a result, IIoT infrastructures are increasingly susceptible to sophisticated cyberattacks, including DoS/Distributed Denial-of-Service (DDoS), ransomware, botnets, advanced persistent threats (APTs), malware infiltration, spoofing, Sybil attacks, sinkhole attacks, session hijacking, selective forwarding, and adversarial manipulation. The impact of such attacks on the operational aspects of the systems goes beyond the compromise of data and can include disruption of industrial processes, critical infrastructure operation, patient safety, energy distribution, and autonomous manufacturing systems (Nandanwar and Katarya, 2024; Alfahaid et al., 2025; Blali et al., 2025).

Traditional security architectures primarily rely on known attack types or static behavioral expectations, which makes them less effective at detecting zero-day attacks, stealthy intrusions, adaptive adversarial behavior, and new attack patterns. Moreover, industrial environments face other operational challenges, such as encrypted traffic analysis, high-dimensional network telemetry, distributed edge deployment, low-latency security requirements, heterogeneous communication protocols, and severe class imbalance in intrusion datasets (Anis et al., 2025). These constraints have propelled the shift toward artificial intelligence (AI)-based cybersecurity architectures that can adaptively detect threats, perform contextual traffic analysis, automatically identify anomalies, and provide intelligent cyber defense.

Machine learning was an early paradigm in IIoT intrusion detection, as it could detect statistical relationships in structured network traffic data. Random Forest (RF), Support Vector Machine (SVM), Decision Tree (DT), k-Nearest Neighbors (KNN), and XGBoost have been widely used to classify structured industrial traffic into a binary or multiclass attack classification. However, traditional machine-learning models require explicit feature engineering and struggle to capture the evolution of temporal attacks and the complex sequential communication patterns of modern-day industrial cyberattacks (Nuaimi et al., 2023).

Deep learning architectures have been extensively employed in the domain of IIoT cybersecurity research to address these challenges. The effective use of Convolutional Neural Networks (CNNs) has significantly improved automated feature extraction from high-dimensional traffic data, while recurrent structures, like Long Short-Term Memory (LSTM) and Gated Recurrent Unit (GRU) networks, have facilitated effective modeling of temporal dependencies for sequential anomaly analysis. To improve spatiotemporal intrusion detection, hybrid CNN-LSTM and attention-driven architectures have been developed to jointly model local traffic patterns and evolving attack behavior. Recently, transformer architectures and graph neural networks (GNNs) have shown promise in the field of contextual cybersecurity intelligence, employing self-attention mechanisms and modeling relationships among entities in distributed industrial environment (Du et al., 2026; Cui et al., 2024; Fraihat et al., 2026).

At the same time, with the growing fragility of industrial data, federated learning has become a new trend in the development of IDSs that can learn together without sharing raw data with a central server. At the same time, as decisions in industrial cybersecurity demand more than just black-box predictions, explainable AI (XAI) has come to the forefront. Furthermore, intelligent industrial cybersecurity is getting new features such as adversarial robustness, continual learning, lightweight edge intelligence, digital twin security simulations, blockchain-based cybersecurity, and autonomous self-healing.

While there has been a significant amount of research focused on AI-enabled cybersecurity in IoT and IIoT, various challenges remain unaddressed. Although many existing methods have demonstrated high detection accuracy on well-known datasets like CICIoT2023, TON-IoT, Edge-IIoTset, UNSW-NB15, CICIoMT2024, and Bot-IoT, these methods are still not widely applicable in real-world industrial scenarios due to the lack of explainability, high computational complexity, strong ability to resist adversarial attacks, high detection rate for stealthy attacks, and minority class attacks. Furthermore, the evaluation is mainly carried out with fixed reference traffic and test scenarios, and there is a lack of evidence of cross-domain generalization, non-IID traffic distributions, concept drift, dynamically changing operational environments, and heterogeneous industrial infrastructures.

Hence, it is important to perform a thorough and critical survey of intelligent cybersecurity architectures for IIoT environments to systematically highlight the latest developments, datasets, attack models, AI-based intrusion detection, deployment challenges, and future research directions. Unlike the previous surveys, which focused on individual learning paradigms or stand-alone intrusion detection methods, this review provides a comprehensive analysis of the latest developments regarding machine learning, deep learning, hybrid intelligence, transformer architectures, graph neural networks, federated learning, explainable AI, reinforcement learning, and trustworthy security protocols for IIoT systems.

The principal contributions of this review are summarized as follows:

- A comprehensive analysis of key cyber threats across the perception, network, and application layers of IIoT environments.
- An in-depth analysis of popular IIoT cybersecurity datasets, their properties, limitations, attack spectrum, and operational relevance.
- A systematic review of AI-based cybersecurity architectures like machine learning, deep learning, hybrid intelligence, transformer-based models, graph neural networks, federated learning, reinforcement learning, and explainable AI systems.
- A comparison of the scalability, explainability, computational complexity, adversarial robustness, privacy preservation, and deployment feasibility of the existing intrusion detection systems.
- A critical discussion of open research challenges such as stealthy attack detection, edge deployment constraints, non-IID federated learning, concept drift, adversarial vulnerability, and trustworthy AI integration.
- Identification of emerging research directions, including explainable federated transformers, continual learning, lightweight edge-native IDSs, digital twin-enabled cybersecurity, self-healing defense systems, and foundation-model-driven cyber intelligence.

### 1.1 Research questions

The systematic review was guided by four research questions formulated to establish a coherent analytical relationship among the IIoT threat landscape, available cybersecurity datasets, AI-driven detection paradigms, operational constraints, and emerging research directions.

**RQ1:** The principal cybersecurity threats affecting contemporary IIoT environments, and how effectively do existing AI-driven approaches address these behavioral and architectural characteristics.

**RQ2:** Identification of cybersecurity datasets for IoT and IIoT available in public domains, and best evaluation practices needed for intrusion detection.

**RQ3:** Highlight the AI-driven IIoT cybersecurity approaches, from conventional machine learning to hybrid deep learning models with transformers, GNNs, federated learning, reinforcement learning, and explainable AI.

**RQ4:** Identify the research gaps for intrusion detection to design and develop a robust, privacy-preserving, and edge-deployable IIoT cybersecurity solutions with emerging research directions and limitations.

The research questions are addressed throughout the review as follows. RQ1 is examined in Section 3 through the analysis of the IIoT threat landscape and AI-based mitigation strategies. RQ2 is addressed in Sections 4 and 5 through the review of publicly available cybersecurity datasets and evaluation methodologies. RQ3 is covered in Sections 2 and 6 by analyzing AI-driven intrusion detection approaches ranging from conventional machine learning to emerging AI paradigms. RQ4 is addressed in Sections 7 and 8 through the discussion of research gaps, deployment challenges, and future research directions.

### 1.2 Research methodology

To facilitate a clear and systematic literature synthesis of AI-enabled cybersecurity in Industrial Internet of Things (IIoT) environments, this review employed a systematic approach. The review process included research question formulation, systematic literature search, application of derived eligibility criteria, selection of studies according to PRISMA, methodological appraisal, data extraction, and evidence synthesis. The literature search took place from January to March 2026, primarily focusing on English language publications from 2020 to 2026. Additional sources, including seminal publications cited for foundational concepts, key datasets, architectures, methodological frameworks, or standards, were used where necessary for contextual support but were not included in the 150-study systematic evidence corpus.

#### 1.2.1 Information sources and search strategy

The systematic literature search was carried out from January to March 2026 to identify the peer-reviewed papers on the use of AI in cybersecurity in IoT and IIoT contexts. The main search focused on English-language publications from 2020 to 2026. The main bibliographic and publisher databases used were IEEE Xplore, Scopus, Web of Science, ScienceDirect, SpringerLink, ACM Digital Library, and Wiley Online Library. Google Scholar and the MDPI publication platform were used for supplementary searches to cover potential missing studies and enrich coverage to capture any potentially relevant studies that had not been included in the main searches. Eligibility was assessed based on full-text publications that could be accessed via open-access or institutional subscription. The search strategy included the following keywords in the four main dimensions of IoT/IIoT environments, cybersecurity and intrusion detection, artificial intelligence and learning-based detection, and emerging distributed, explainable, and trustworthy intelligence paradigms. The main boolean search structure was created in the following way:

(“Industrial Internet of Things” OR “IIoT” OR “Industrial IoT” OR “Internet of Things” OR “IoT”) AND (“cybersecurity” OR “cyber security” OR “intrusion detection” OR “intrusion detection system” OR “IDS” OR “cyber threat” OR “attack detection” OR “anomaly detection”) AND (“artificial intelligence” OR “machine learning” OR “deep learning” OR “CNN” OR “LSTM” OR “GRU” OR “transformer” OR “graph neural network” OR “GNN” OR “federated learning” OR “reinforcement learning” OR “explainable AI” OR “XAI”).

The following terms were also used to search selectively, such as edge intelligence, adversarial machine learning, concept drift, digital twins, large language models, foundation models, multimodal AI, autonomous cyber defense, IoT/IIoT cybersecurity datasets. The search words or phrases were modified to fit the indexing system and search-field characteristics of each database but still retained the general idea of the search.

#### 1.2.2 Eligibility criteria

The literature that was selected was consistent with the scope and research questions of the review using predefined eligibility criteria. Eligible studies were those published between 2020 and 2026, written in English, and falling into one of the following categories: (i) peer-reviewed research articles, conference papers, and/or methodologically relevant review articles; (ii) studies that considered cybersecurity, intrusion detection, anomaly detection, attack detection, or closely related security problems in IoT, IIoT, industrial cyber-physical, or connected industrial environments; and (iii) studies that provided substantive evidence related to AI-driven detection techniques, cybersecurity datasets, evaluation methodologies, privacy, explainability, adversarial robustness, or operational deployment.

Studies were not included if they were duplicated, were outside the scope of the research needed for this study, were not available in English, lacked sufficient methodological or technical information for the types of synthesis needed, or were not substantial in cybersecurity but only featured AI. Eligibility assessment required full-text access via open access or institutional subscription. For those seminal papers prior to 2020, which were cited as the basis for foundational datasets, architectures, or concepts presented in the review, they were kept selectively if necessary to trace the origins of those concepts. The publications were used as references instead of evidence of the time span of the systematic search.

#### 1.2.3 Study selection and PRISMA workflow

**Figure 1:**
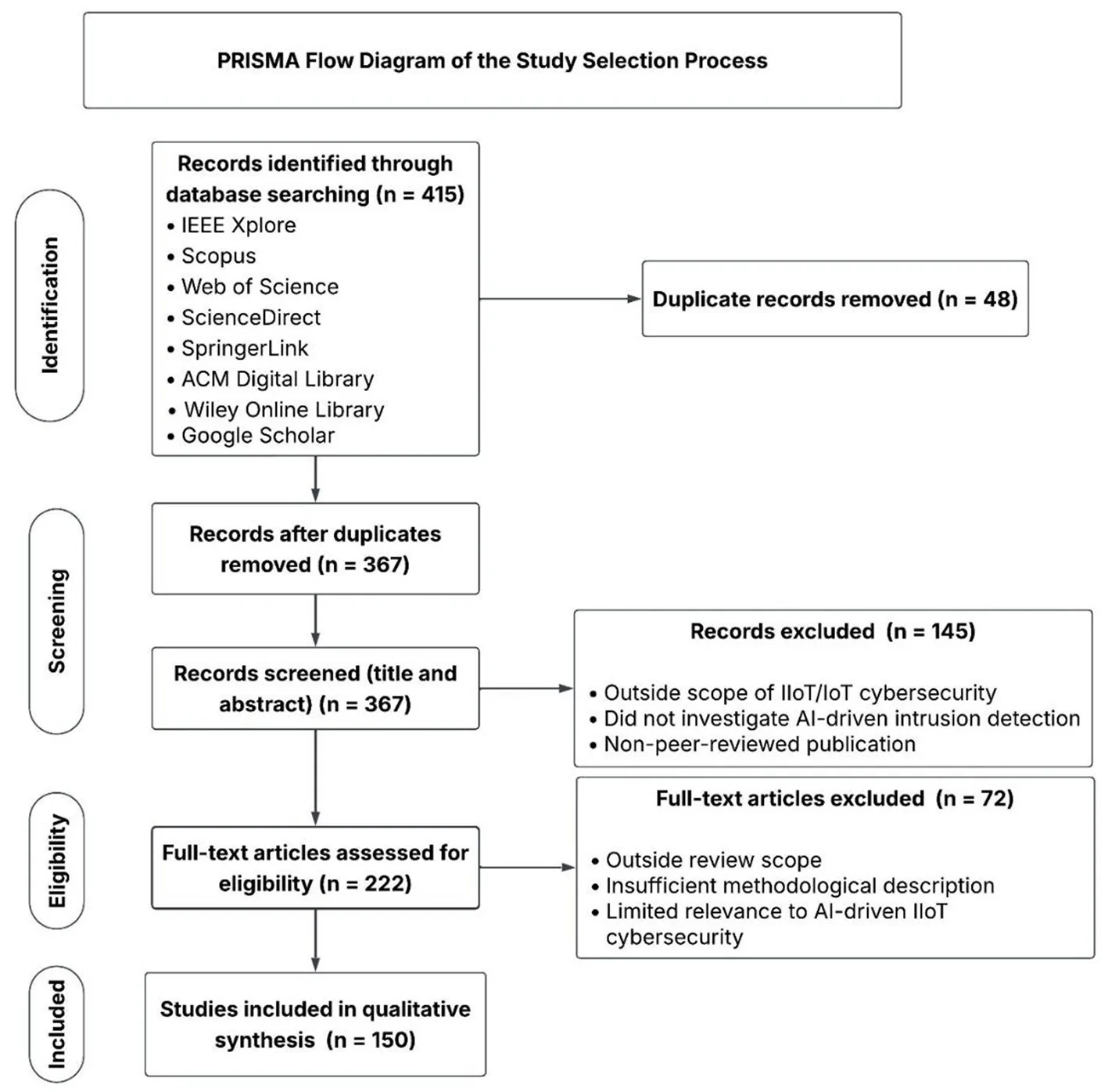
PRISMA-based study-selection workflow for AI-driven IIoT cybersecurity.

The selection of studies was done in a PRISMA workflow: identification, duplicate removal, title and abstract screening, eligibility assessment of full text, and final inclusion. A total of 415 potentially relevant documents were found in the selected databases and via supplementary scholarly literature searches. The retrieved records were consolidated, and 48 duplicate records were removed, which resulted in 367 unique records for title and abstract screening. The titles and abstracts of the 367 records were screened for inclusion, and those that met the scope and eligibility requirements were reviewed. In this phase, we excluded 145 records for failing to meet the preliminary eligibility criteria and included 222 records for full-text evaluation. The full texts of these articles were then reviewed in light of the preset inclusion and exclusion criteria. Full-text assessment eliminated another 72 articles. As a result, 150 studies were selected for inclusion in the systematic evidence corpus used for the review synthesis. These studies formed the primary evidence base for the analysis of IIoT cyber threats, cybersecurity datasets, evaluation practices, AI-based intrusion detection methods, deployment constraints, research gaps, and emerging research directions. The reference list additionally includes supporting sources cited for foundational concepts, dataset descriptions, methodological frameworks, standards, and broader contextual purposes; these references were not counted among the 150 studies included through the PRISMA workflow. The entire PRISMA-based study-selection workflow is illustrated in Figure 1.

#### 1.2.4 Data extraction and methodological appraisal

The eligible studies were reviewed, and the information pertinent to the research questions was systematically extracted. For studies using AI capabilities, the attributes extracted comprised the publication year, the application context, the dataset, the learning paradigm, the model architecture, the reported evaluation metrics, the principal methodological contribution, and the identified limitations. Dataset-oriented studies were examined with respect to device environments, communication protocols, attack taxonomy, collection methods, feature representation, learning suitability, and strengths and drawbacks. Further, if reported, information about computational requirements, explainability, privacy preservation, adversarial robustness, scalability, and deployment feasibility was also taken into consideration.

Selected literature was critically appraised based on the clarity of the research objective, the quality of the description of the dataset and experimental method, the transparency of the proposed methodology, the suitability of the evaluation metrics, the availability of a comparative evaluation, and the relevance of the literature to the formulated research questions. Reported classification accuracy was not considered sufficient proof of methodological superiority because benchmark performance does not necessarily reflect generalization, robustness, computational feasibility, and operational effectiveness in heterogeneous IIoT environments.

### 1.3 Comparison with existing surveys and novelty of the present review

Several surveys have examined cybersecurity and intrusion detection in IoT environments from different methodological perspectives. However, their scope varies considerably with respect to the threat landscape, learning paradigms, privacy-preserving mechanisms, explainability, and systematic review methodology. Table 1 positions the present review against representative existing surveys and highlights its broader IIoT-oriented scope.

**Table 1:** Novelty of the proposed review in comparison with the recent surveys.

Survey	Domain scope	Primary coverage	XAI/ interpretability	FL	Emerging AI/advanced paradigms	Systematic review framework
Swessi and Idoudi (2022)	General IoT security	Security requirements, threat taxonomy, architecture-level attacks, and emerging countermeasures	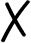	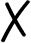	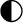	Not reported
Santhosh Kumar et al. (2023)	IoT intrusion detection	ML-based IDS, ML/DL techniques, intrusion-detection architectures and security mechanisms	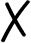	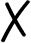	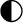	Not reported
Omar and Soudan (2023)	RPL-based IoT security	Sinkhole attacks; anomaly-, signature-, specification-, trust-, ranking-, and ML-based detection	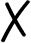	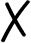	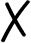	Not reported
Alfahaid et al. (2025)	General IoT security	ML, DL, EL, RL, FL, TL, privacy-preserving and edge-oriented security	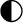	✓	✓	Structured methodology
Anis et al. (2025)	Network intrusion detection	DL-based NIDS, generative models, transformers, CNN/DNN, and hybrid/deployment-oriented architectures	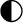	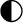	✓	Structured literature synthesis
Present review	IIoT cybersecurity	ML, DL, hybrid AI, transformers, GNNs, FL, RL, XAI, and emerging AI paradigms	✓	✓	✓	PRISMA-based + 4 RQs

✓ Indicates explicit coverage; 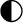 indicates limited or partial coverage; 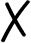 indicates that the topic is not substantively addressed. FL, federated learning; XAI, explainable artificial intelligence.

As shown in Table 1, existing surveys have made substantial contributions to IoT security from complementary perspectives, including architecture-oriented threat taxonomies, machine learning-based intrusion detection, attack-specific mitigation, and deep learning-based network intrusion detection. More recent surveys have further expanded this scope toward federated learning, transfer learning, privacy preservation, interpretability, and edge-oriented security. Nevertheless, comparative analyses indicate that these dimensions are generally examined within broader IoT security, specific attack scenarios, or individual methodological paradigms rather than jointly synthesized from an IIoT-oriented operational perspective. The present review therefore integrates architectural threat analysis, cybersecurity datasets, evaluation practices, conventional and emerging AI paradigms, privacy-preserving learning, explainability, adversarial robustness, edge-deployment constraints, and cross-domain generalization within a unified IIoT cybersecurity framework. Furthermore, the review employs explicit research questions and a PRISMA-based study-selection process to systematically connect the identified evidence with open challenges and emerging research directions toward trustworthy and deployable AI-driven IIoT cybersecurity.

**Figure 2:**
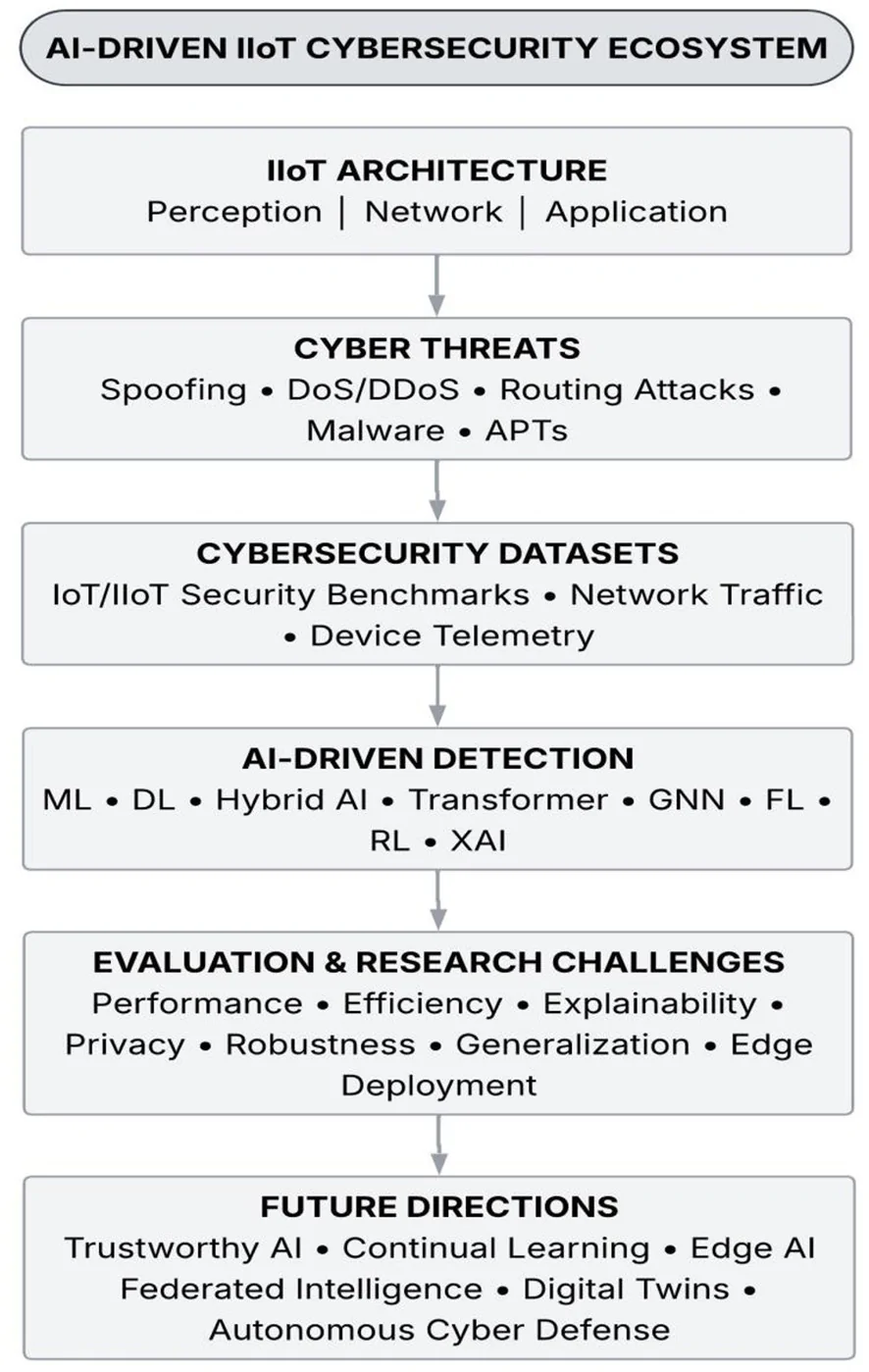
Framework of the AI-driven IIoT cybersecurity ecosystem.

The integrated scope of the present review is conceptualized in Figure 2, which establishes the analytical relationship among the IIoT architectural layers, corresponding cyber threats, AI-driven detection paradigms, cybersecurity datasets, evaluation criteria, and the operational challenges that motivate emerging research directions. Rather than treating these components independently, the framework illustrates their interdependence within an end-to-end IIoT cybersecurity ecosystem, thereby providing the conceptual basis for the subsequent sections of this review.

The subsequent sections of this manuscript are organized as follows. Section 2 presents a comprehensive analysis of recent studies on AI-driven intrusion detection and cybersecurity measures within the IIoT context. Section 3 examines major IIoT cyber threats and corresponding AI-driven mitigation strategies across different architectural layers. Section 4 analyzes publicly available IoT and IIoT cybersecurity datasets, including their characteristics, strengths, limitations, and suitability for AI-driven intrusion detection. Section 5 discusses the principal evaluation metrics used to assess intelligent intrusion detection and cybersecurity systems. Section 6 examines emerging AI-driven techniques for cyber threat detection. Section 7 identifies the principal open research challenges and gaps in AI-driven IIoT cybersecurity. Section 8 provides an integrated discussion and presents future research directions toward intelligent, explainable, federated, adaptive, and trustworthy IIoT security systems. Finally, Section 9 concludes the manuscript.

## 2 Literature review

As IoT networks grow in scope and complexity, novel threats are arising, becoming increasingly intricate and intelligent, rendering traditional intrusion detection systems (IDSs) increasingly ineffective. This section presents a detailed and thematic overview of 32 recent publications (2022–2025) on intrusion and anomaly detection, adversarial robustness, and cyber threat classification in IoT and IIoT applications. The reviewed papers are classified in the following categories: (i) transformer-based and attention-driven architectures, (ii) hybrid CNN-LSTM and spatial-temporal models, (iii) graph neural network approaches, (iv) federated learning frameworks, (v) reinforcement learning-based IDS, (vi) machine learning and ensemble methods, and (vii) adversarial robustness and explainability.

### 2.1 Transformer-based and attention-driven architectures

Transformer models have successfully identified long-term relationships in linear data and have been progressively utilized in network intrusion detection systems. Several studies support the superiority of transformer and multi-head self-attention mechanisms over traditional recurrent and convolutional networks.

Akuthota and Bhargava (2025) demonstrated that lightweight transformer architectures that combine multi-head self-attention and focal loss led to continuous improvements in multiclass intrusion detection with moderate complexity appropriate for resource-limited IoT applications. Similarly, Benitha Christinal and Ameelia Roseline (2025) combined transformer learning with TB-SMOTE balancing and attention-driven transfer learning to enhance adaptation to previously unseen attacks and reduce training overhead and memory usage by optimizing the learning process with transfer learning and quantization-aware deployment strategies.

Recent research also continues the attention-driven cybersecurity research by using hybrid contextual representation learning. To enhance contextual traffic representation in Edge-IIoTset environments, Ishtiaq et al. (2025) introduced a novel design, CST-AFNet, that combines multi-scale convolutional learning, bidirectional GRU, temporal attention, and channel attention. Similarly, Sasi et al. (2024) employed residual CNN-LSTM architectures, self-attention, and SHAP-based explanations of features to enhance generalization across different datasets and interpretability of different heterogeneous IoT datasets. Vellela et al. (2025) also showed that integrating semantic embedding techniques with a self-attention-enhanced BiLSTM architecture was helpful for representing the contextual threats in communication environments of the Industry 4.0 revolution.

### 2.2 Hybrid CNN-LSTM and spatial-temporal models

Recent research by Polat et al. (2025) has shown that hybrid CNN-LSTM networks integrating multi-head self-attention and imbalance-aware optimization can enhance malware classification and intrusion detection in IoT-based SCADA systems. However, the framework demonstrated poor detection performance for stealth-oriented application-layer attacks, such as Structured Query Language (SQL) injection, Cross-Site Scripting (XSS), and backdoor malware, despite achieving nearly perfect results in volumetric attacks. Likewise, Zahid and Bharati (2025) used the CNN-BiLSTM architecture and adopted the LASSO feature selection method, along with lightweight optimization approaches, to make edge deployment more feasible in real time and also reduce model overfitting and complexity. Given the complexity and sensitivity of the Internet of Medical Things (IoMT) communication environment, hybrid spatial–temporal architectures are becoming more popular in healthcare-oriented cybersecurity research. Babar et al. (2025) combined BiLSTM-CNN architectures and SDN with fog nodes in fog environments for intrusion detection and fog-node selection in healthcare IoT systems. Similarly, Berguiga et al. (2025) further extended hybrid deep learning intrusion detection to RPL-based IoMT communication within distributed fog-edge-cloud deployment architectures. Additionally, Shaikh et al. (2025) integrated CNN-LSTM models with Deep Q-Network (DQN) and Proximal Policy Optimization (PPO) reinforcement learning algorithms to enhance the adaptive intrusion detection capabilities in the context of CICIoMT2024 healthcare environments. Recent works also focus on the potential of ensemble learning and optimization-aware pre-processing in order to tackle class imbalance and minority attack detection. In a highly imbalanced BoT-IoT environment, Zhang (2025) demonstrated that the weighted ensembles of CNN-GRU, CNN-LSTM, and DNN architectures performed better on minority attack detection while retaining real-time inference capability on the Raspberry Pi hardware. Soliman et al. (2023) highlighted the GRU architecture with dimensionality reduction and imbalance-aware data preprocessing was effective in an IIoT intrusion detection system using TON-IoT datasets. Similarly, Cao et al. (2025) demonstrated that telemetry-based preprocessing and cyclic encoding of the temporal dimension enhance classification capability for time-sensitive patterns of industrial attacks.

### 2.3 Graph neural network approaches

Graph Neural Networks (GNNs) provide a principled approach to capturing the structural and temporal relationships within networks, especially IoT networks. Several works propose new GNN architectures that can model dynamic inter-device relationships for anomaly and fault detection. Recent cybersecurity graph-based research has shifted to dynamic graph learning, graph attention mechanisms, causality-inspired dependency modeling, and energy-efficient anomaly detection. Huang et al. (2025) introduced a hybrid GAT-LSTM framework designed to address quasi-dynamic graph structures in symbiotic IoT contexts, significantly improving intermittent fault diagnosis and temporal anomaly representation. Similarly, Liu et al. (2024) proposed a temporal dynamic graph neural network (TodyNet) that can learn dynamic inter-variable dependencies without requiring predefined graph structures, enhancing adaptability for multivariate time-series classification. Causal learning and efficient graph optimization are also becoming more popular topics in cloud-IoT security research, and both are inspired by graph structures. To improve detection in cloud-enabled IoT systems, Begum et al. (2025) combined a graph learning algorithm based on Granger causality with EfficientcoviNet architectures and advanced optimization techniques to increase the scalability of the intrusion detection system. Similarly, Guo et al. (2024) suggested an adaptive mode switching energy-efficient graph neural framework (AMSWGNN) that leverages subgraph learning to efficiently utilize limited computational resources while maintaining competitive performance in anomaly detection tasks in low-power IoT settings.

### 2.4 Federated learning frameworks for IoT security

Federated learning (FL) addresses critical privacy concerns in distributed IoT environments by enabling model training without raw data exchange. Multiple studies explore FL-based IDS designs with varying aggregation strategies, model architectures, and optimization schemes. Recent federated intrusion detection studies increasingly integrate secure communication protocols, optimization-aware aggregation, lightweight local learning, and distributed anomaly analysis. Bensaid et al. (2025) proposed 2FIDS, a fog-assisted federated intrusion detection framework integrating LSTM-based local learning, secure TLS/gRPC communication, median aggregation, and quantization-aware optimization to reduce communication overhead within distributed smart-home environments. Karunamurthy et al. (2025) further combined federated learning with chimp optimization-based feature selection to improve intrusion detection capability for MQTT-oriented IoT communication. Comparative analyses of supervised and unsupervised federated architectures additionally demonstrate the growing importance of distributed collaborative intelligence within heterogeneous IoT ecosystems. Olanrewaju-George and Pranggono (2025) systematically compared federated deep neural networks and federated autoencoders across multiple IoT devices, showing that federated architectures considerably improve privacy-preserving intrusion detection while simultaneously reducing false positive rates in distributed environments.

### 2.5 Reinforcement learning-based approaches

To enhance adaptive intrusion detection capability in large-scale BoT-IoT environments, Khayat et al. (2025) proposed an RL-IDS that combines deep Q-network learning, hybrid optimization-aware feature selection, autoencoder-based state representation, and deep feature fusion. The framework consistently surpassed conventional CNN, DNN, LSTM, and GAN-DRL baselines and was also shown to learn rapidly when encountering changing attack patterns. Wang and Aouf (2024) introduced an explainable adversarial reinforcement learning framework that combines PPO optimization and saliency-based adversarial detection methods to improve adversarial robustness in safety-critical intelligent environments.

### 2.6 Machine learning and ensemble methods

While deep learning methods are frequently emphasized in recent publications, traditional and ensemble ML methods are still competitive and are appealing for IoT network traffic data in tabular format, where these methods are interpretable, efficient to train, and have a light footprint. To achieve this, Shahin et al. (2024) conducted the most comprehensive assessment to date of over 25 ML, DL, and Deep Hybrid Learning (DHL) algorithms for detecting IoT botnets, using the N-BaIoT dataset across seven commercial IoT devices to identify Mirai and Gafgyt botnets. Shahin et al. (2022) proposed two DHL models with ALSTM-FCN and XGBoost and AdaBoost for attack detection for seven distinct kinds of IoT devices in the TON-IoT dataset. The attention mechanism guided learning to the most important information contained in the time-series segments, and the hybrid ML-DL architecture used deep feature extraction combined with strong ensemble classification. Likewise, Ismail et al. (2025) demonstrated the excellent generalization ability and resource efficiency in the TON-IoT, WUSTL-IIoT-2021, and Edge-IIoTset environments for lightweight ensemble methods. Another interesting observation by Hakami et al. (2025) was that the efficacy of Random Forest is significantly better than recurrent architectures in cases of tabular IDS, with significantly lower inference latency appropriate for real-time IoT implementation. Similarly, Alsubaei (2025) combined an enhanced XGBoost algorithm featuring an improved sequential neural network (OSNN) tuned by five-fold cross-validation using grid and randomized hyperparameter search and tested on NSL-KDD, UNSW-NB15, and CIC-IDS2017. Benmalek and Seddiki (2025) used a wrapper feature selection technique, namely Particle Swarm Optimization (PSO), with a Random Forest estimator on the real-time RT_IoT2022 dataset.

### 2.7 Adversarial robustness and explainability

With the growing use of DL-based IDS models in critical infrastructure, the vulnerability of these models to adversarial attacks and their lack of interpretability have become critical. Adversarial robustness improvement and integration of explainability are discussed in several works explicitly. Shtayat et al. (2023) integrated SHapley Additive exPlanations (SHAP) and Local Interpretable Model-Agnostic Explanations (LIME) into ensemble learning systems to attain both global and local explainability of IIoT intrusion detection systems (IDS), enhancing analysts' trust and forensic interpretability. Likewise, Eleftheriadis et al. (2024) proposed a comprehensive approach to making DNNs adversarially robust, including the Adversarial Robustness Evaluation Benchmark (AREB) with four popular attacks: PGD (L∞, white-box), Carlini and Wagner L2 (white-box), Elastic Net L1 (white-box), and HopSkipJump L2 (black-box). Ragab et al. (2025) proposed an ensemble of CNN and Deep Belief Network (DBN) with Honey-Badger Algorithm (HBA) feature selection and Lotus Effect Optimization Algorithm (LEOA) hyperparameter optimization using the TON-IoT dataset with 10 attack classes. Recent work also investigates new hybrid architectures based on computer vision and contextual representation learning. Wasswa et al. (2025) proposed ResDNViT, a hybrid Residual Dense Network (ResDenseNet) and Vision Transformer (ViT), to perform contextual attack analysis of NetFlow traffic by constructing an image-like representation of each NetFlow record. The framework also achieved excellent generalization performance on various IoT datasets, including unseen device categories, underscoring the need for cross-domain representation learning in intelligent cybersecurity. To consolidate the evidence discussed across Sections 2.1–2.7, Table 2 provides a study-level comparison of the reviewed literature in terms of datasets, methodological architectures, reported performance, principal strengths, and identified limitations.

**Table 2:** Summary of reviewed studies on AI-driven intrusion detection and anomaly detection in IoT/IIoT environments.

References	Dataset	Methodology	Performance metrics	Key strengths	Limitations
Akuthota and Bhargava (2025)	NSL-KDD, UNSW-NB15	Transformer-based IDS with multi-head self-attention and focal loss	NSL-KDD: ACC: 99.52; F1: 99.53. UNSW-NB15: ACC: 99.97; F1: 99.96	Lightweight high-performance transformer IDS suitable for resource-limited IoT	No real-time edge deployment or live traffic validation
Benitha Christinal and Ameelia Roseline (2025)	InSDN, SDNFlow	TMSAM transformer framework with TB-SMOTE and attention-driven transfer learning (ATL)	ACC: 99.40; P: 99.42; R: 99.38; F1: 99.40	Effective class imbalance handling and rapid transfer learning adaptation	High transformer computational and memory requirements
Ishtiaq et al. (2025)	Edge-IIoTset	CST-AFNet with multi-scale CNN, BiGRU, temporal and channel attention	Multiclass: ACC: 99.97; macro-F1: 99.37. Binary: ACC: 100.00	Dual-attention framework with strong rare-attack detection capability	Computational overhead and lack of adversarial evaluation
Sasi et al. (2024)	CIC-BCCC-NRC_ TabularIoTAttack-2024 (9 IoT datasets)	Self-attention 1D-CNN-LSTM with residual blocks and SHAP-based feature selection	CICIoMT2024: ACC: 99.88; F1: 99.94. TON-IoT-2021: F1: 99.99. Edge-IIoTset: ACC: 33.30	Strong cross-dataset generalization with interpretable feature compression	Severe degradation on Edge-IIoTset and binary-only classification
Vellela et al. (2025)	CIC-IDS2018	SA-BiLSTM with GloVe embeddings, self-attention, PCA, SCOA, and GLGOA	ACC: 99.30; P: 99.00; R: 99.00; F1: 99.30	Advanced semantic feature learning with optimized BiLSTM intrusion detection	Single-dataset evaluation and potential overfitting due to oversampling
Polat et al. (2025)	CICIoT2023	Hybrid CNN-LSTM with multi-head self-attention, partial-SMOTE, weighted loss	ACC: 99.23; AUC: 0.9992	Strong malware and volumetric attack detection for IoT-SCADA systems	Poor detection of stealthy application-layer attacks
Zahid and Bharati (2025)	KDDCup99, NSL-KDD, CIC-IDS2017	Hybrid CNN-BiLSTM with LASSO feature selection and SMOTE balancing	KDDCup99: ACC: 99.90. NSL-KDD: ACC: 99.84. CIC-IDS2017: ACC: 97.97; AUC: 0.99–1.00	Lightweight real-time IDS with strong spatial-temporal learning capability	Lower detection for rare attacks and no evaluation on modern IoT-specific datasets
Babar et al. (2025)	CICDDoS2019	SDN-driven hybrid framework using BiLSTM for attack detection and CNN for fog node selection	ACC: 99.59; P: 99.44; R: 99.15; F1: 99.34	Joint optimization of security and QoS with ultra-low latency and efficient fog management	Scalability degradation with large fog networks and no explainable AI integration
Berguiga et al. (2025)	CIC-DDoS2019	HIDS-RPL hybrid IDS using CNN-LSTM for RPL-based IoMT intrusion detection	ACC: 99.87; P: 98.50; R: 98.64; F1: 98.54	Effective RPL-aware DDoS detection with fog-tier distributed architecture	Limited to DDoS attacks and lacks explainability mechanisms
Shaikh et al. (2025)	CICIoMT2024	HCLR-IDS using CNN-LSTM with DQN and PPO reinforcement learning	Binary: ACC: 99.58; P: 99.53; R: 99.83; F1: 99.57	Adaptive RL-driven IDS with strong binary intrusion detection performance	Reduced macro-level multiclass performance due to severe class imbalance
Zhang (2025)	BoT-IoT	Ensemble IDS combining CNN-GRU, CNN-LSTM, and DNN with weighted averaging	ACC: 99.98; P: 99.28; R: 99.80; F1: 99.54	Real-time edge-capable IDS with strong rare-class detection improvement	Single-dataset evaluation and increased ensemble computational complexity
Soliman et al. (2023)	TON-IoT	Deep learning IDS using GRU, LSTM, and Bi-LSTM with SVD and SMOTE	Binary: ACC: 99.99. Multiclass: ACC: 99.98; F1: 100.00	Near-perfect intrusion detection performance using GRU with balanced data engineering	High computational training cost and lack of interpretability
Cao et al. (2025)	TON-IoT	IDS using KNN, CART, RF, CNN, LSTM, and DNN with temporal cyclic encoding and SMOTE+Tomek balancing	Binary: CART and RF achieved 100% across all evaluation metrics. Multiclass (DNN): ACC: 98.71; F1: 98.74	Realistic merged IIoT-network dataset handling with temporal encoding	Potential overfitting and absence of explainable AI analysis
Huang et al. (2025)	WADI	IFDGAT-LSTM integrating graph attention network and LSTM with quasi-dynamic graph modeling	P: 99.58; R: 57.82; F1: 73.00	Joint spatial-temporal anomaly modeling for dynamic IoT environments	Moderate recall and evaluation limited to the WADI dataset
Liu et al. (2024)	26 UEA MTSC benchmark datasets	TodyNet dynamic graph neural network with dynamic graph transform, DyGIN, temporal convolution, and temporal graph pooling	Average ACC: 73.00	Learns dynamic temporal dependencies without predefined graph structures	O(N^2^) graph complexity and restricted to equal-length time series
Begum et al. (2025)	CIC-IDS2017	DNS-GCIGNNENet-CIOTD integrating GCIGNN, EfficientcoviNet, and LNSMO optimization	Relative improvement: ACC: +25%; P: +26%; R: +28%; F1: +28%	Linear time complexity with robust graph-based cloud IoT security	Validation restricted to CIC-IDS2017
Guo et al. (2024)	SWaT, PSM, MSL, SMAP	EGNN energy-efficient GNN with subgraph generation and adaptive mode switching	SWaT: F1: 77.33. PSM: F1: 91.05. MSL: F1: 84.92. SMAP: F1: 89.21	Energy-efficient anomaly detection with cloud-edge collaboration	Scalability dependent on accurate graph learning
Bensaid et al. (2025)	BoT-IoT, TON-IoT, MQTTset	2FIDS federated LSTM IDS with fog-assisted FL, median aggregation, TLS/gRPC, quantization and compression	BoT-IoT: ACC: 96.50; F1: 96.89. TON-IoT: ACC: 96.49; F1: 95.32. MQTTset: ACC: 86.36; F1: 86.36	Privacy-preserving federated IDS with strong scalability and communication efficiency	Lower MQTTset performance; no non-IID or real-world deployment validation
Karunamurthy et al. (2025)	MQTTset	Federated learning CNN with chimp optimization feature selection	ACC: 95.59; P: 94.79; R: 93.30; F1: 94.04	Privacy-preserving federated IDS with optimized feature reduction	Vulnerable to poisoned model updates and tested only on MQTTset
Olanrewaju-George and Pranggono (2025)	N-BaIoT	Federated autoencoder and deep neural network using FedAvgM	FL-DNN: ACC: 90.39; P: 99.99; R: 90.10; F1: 93.12	Comparative FL evaluation of supervised and unsupervised DL IDS models	Scalability and heterogeneous device validation not explored
Khayat et al. (2025)	BoT-IoT	RL-IDS integrating DQN reinforcement learning with CNN, LSTM, PCA, GAO, and CL-OOA feature optimization	ACC: 99.80; P: 99.99; R: 99.79; F1: 99.89; FPR: 0.0019	Adaptive RL-based IDSs with optimized feature fusion and low computation time	High hyperparameter sensitivity and limited cross-dataset validation
Wang and Aouf (2024)	CARLA Simulator	Explainable adversarial DRL using PPO, FGSM/BIM attacks, saliency-based CNN detector	ACC: 99.38	Robust and explainable adversarial defense framework	Simulation-only evaluation without real-world validation
Shahin et al. (2024)	N-BaIoT	Benchmarking of >25 ML, DL, and deep hybrid learning models including RF, XGB, CNN, LSTM, GRU, CNN-LSTM-XGB, FCN-LSTM-Adaboost	ML ensemble models: nearly 100% ACC, P, and R for Mirai and Gafgyt detection. DL models: ACC: 95.00–99.90	Comprehensive device-wise benchmarking with hybrid learning evaluation	Limited to Mirai and Gafgyt botnets and seven N-BaIoT devices
Shahin et al. (2022)	TON-IoT	Deep hybrid learning using ALSTM-FCN with XGBoost and AdaBoost	P: 95.97; R: 95.68; F1: 95.74	Hybrid DL–ML fusion with attention mechanism improved IIoT attack detection	Lower performance on Modbus device and no real-time deployment validation
Ismail et al. (2025)	TON-IoT, WUSTL-IIoT-2021, Edge-IIoTset	Lightweight IDS using DT, RF, LightGBM, bagging, and stacking	TON-IoT: LightGBM Micro-F1: 97.80. WUSTL-IIoT-2021: stacking micro-F1: ~87.00. Edge-IIoTset: nearly 100% detection for DDoS_UDP and DDoS_ICMP	Lightweight models suitable for resource-constrained IIoT environments	Poor detection for rare attacks such as MITM and backdoor
Hakami et al. (2025)	WSN-DS, UNSW-NB15, CIC-IDS2017	Random forest and LSTM with PCC feature selection and SMOTE balancing	RF: WSN-DS: ACC: 99.71. UNSW-NB15: ACC: 90.17. CIC-IDS2017: ACC: 99.99	Strong generalization across multiple IDS datasets with efficient feature filtering	Rare attack classes removed and no real-time deployment analysis
Alsubaei (2025)	NSL-KDD, UNSW-NB15, CIC-IDS2017	Optimized XGBoost and optimized sequential neural network (OSNN)	Binary: ACC: 99.93; F1: 99.84; MCC: 0.9986. CIC-IDS2017: ACC: 99.53	Extremely low false positive rate with robust multiclass generalization	Weak detection of rare classes such as R2L and high computational demand
Benmalek and Seddiki (2025)	RT_IoT2022	PSO-enhanced IDS using CatBoost, CNN, LSTM, KNN, SVM, and Naïve Bayes	CatBoost: binary: ACC: 99.85. Multiclass: ACC: 99.82	High-accuracy intrusion detection with major reduction in computational cost	Limited adversarial robustness and weaker performance on rare attack classes
Shtayat et al. (2023)	TON-IoT	Explainable ensemble IDS using CNN-1, CNN-2, CNN-3 with SHAP and LIME	Binary: ACC: 99.69; F1: 100.00. Multiclass: ACC: 99.63; F1: 99.50	Integrated explainability using SHAP and LIME with robust CNN ensemble	Increased computational complexity and lack of real-time deployment evaluation
Eleftheriadis et al. (2024)	MNIST, CIFAR-10	Holistic adversarial robustness framework using AREB benchmark and ensemble adversarial training	MNIST: PGD robustness improved from 87.30 to 98.40 after ensemble adversarial training	Comprehensive adversarial robustness enhancement across multiple attack norms	Very high computational cost and evaluation limited to image datasets
Ragab et al. (2025)	TON-IoT	NGCAD-EDLM integrating CNN, DBN, Honey-Badger algorithm, and lotus effect optimization	ACC: 99.21; P: 95.95; R: 94.62; F1: 95.20; MCC: 94.81	Optimized ensemble DL model with fast computation and robust multiclass detection	High training complexity and reduced recall for MITM attacks
Wasswa et al. (2025)	CIC-IDS2017_improved, Bot-IoT, N-BaIoT, CICIoT2022	ResDNViT combining vision transformer encoder with residual dense network using NetFlow patch transformation	CIC-IDS2017: ACC: 99.93. Bot-IoT: ACC: 99.85. CICIoT2022: ACC: 98.42. N-BaIoT: binary and 3-class ACC: 99.99; 10-class ACC: 99.80	Strong generalization to unseen IoT devices with patch-based ViT innovation	Quadratic ViT complexity and high inference cost for edge deployment

ACC, accuracy; P, precision; R, recall; F1, F1-score; AUC, area under the receiver operating characteristic curve; MCC, Matthews correlation coefficient; FPR, false positive rate; FNR, false negative rate. Performance values are reported as percentages (%) except AUC and MCC.

### 2.8 Critical synthesis, research gaps, and taxonomy

The literature reviewed clearly illustrates the evolution of the methods from traditional machine learning and ensemble classifiers to hybrid architectures of spatial-temporal type, attention and transformer models, graph-based learning, federated intelligence, reinforcement learning, and explainable or adversarial-robust architectures. This evolution involves moving from maximizing classification accuracy on a set of structured traffic features to modeling temporal dependency, context, distributed learning, and operational trust. However, none of these paradigms consistently meets the multifaceted demands of detection, computational efficiency, generalization, privacy, robustness, and interpretability. While lightweight ML and ensemble methods still hold promise for structured traffic and resource-constrained deployments (Ismail et al., 2025; Hakami et al., 2025), CNN–LSTM and attention-based models offer superior spatial–temporal representation capabilities, albeit at a higher computational cost (Polat et al., 2025; Sasi et al., 2024). GNNs solve the problem of relational and dynamic dependency modeling but have challenges of constructing graphs and scalability (Liu et al., 2024; Guo et al., 2024), while federated approaches minimize the need for central data but face issues of heterogeneous clients, communication needs, and poisoning risk (Bensaid et al., 2025; Karunamurthy et al., 2025).

A comparison across studies also shows that achieving high benchmark scores does not always mean that the operation is robust. A variety of methodologies have been found to be highly effective in detecting attacks on well-known datasets but may suffer from degraded performance in rare or stealth-oriented attacks, in unseen environments, or with alternative distributions of traffic; e.g., Polat et al. (2025) reported degraded performance in detecting stealth-oriented attacks, while Sasi et al. (2024) indicated that substantial performance degradation occurred on Edge-IIoTset, and Ismail et al. (2025) identified significant challenges in detecting rare attacks. In parallel, explainability, adversarial robustness, and adaptive learning are emerging as features in the XAI and reinforcement-learning paradigms (Shtayat et al., 2023; Khayat et al., 2025), but these properties are not as rigorously studied as detection performance. Overall, the evidence reveals significant and enduring shortcomings in the generalization capabilities across different datasets, minority and stealthy attack detection, edge-oriented computational efficiency, adversarial robustness, explainability, realistic heterogeneous federated evaluation, and real-world deployment validation. Table 3 consolidates these findings into a taxonomy connecting the principal AI paradigms with their security objectives, representative datasets, application domains, comparative strengths, and unresolved limitations.

**Table 3:** Taxonomy and critical comparison of AI-driven cybersecurity approaches (Sasi et al., 2024; Polat et al., 2025; Bensaid et al., 2025; Shtayat et al., 2023).

AI paradigm	Threat/security focus	Representative datasets	Application domain	Comparative strength	Principal limitation
Transformer and attention-based learning	Multiclass intrusion, contextual and complex traffic-pattern detection	NSL-KDD, UNSW-NB15, Edge-IIoTset, CICIoMT2024, CIC-IDS2018	IoT, IIoT, SDN-IoT	Long-range and contextual dependency modeling	Computational and memory overhead; inconsistent cross-dataset performance
Hybrid spatial–temporal DL	DDoS, malware, RPL attacks, general intrusion and minority attacks	CICIoT2023, CICIoMT2024, TON-IoT, BoT-IoT, CIC-DDoS2019	IoT, IIoT, SCADA, IoMT	Joint spatial–temporal feature representation	Model complexity; weaker performance for some rare or stealthy attacks
Graph neural networks	Temporal anomaly, fault and dependency-based detection	WADI, SWaT, PSM, MSL, SMAP, CIC-IDS2017	IoT, cloud-IoT, cyber-physical environments	Explicit relational and dynamic dependency modeling	Graph-learning complexity, scalability, and domain-specific validation
Federated learning	Distributed and privacy-preserving intrusion detection	BoT-IoT, TON-IoT, MQTTset, N-BaIoT	Smart home, IoT, fog-assisted environments	Collaborative learning without centralized raw-data exchange	Non-IID/device heterogeneity, poisoning vulnerability, and deployment validation
Reinforcement learning	Adaptive intrusion detection and adversarial decision-making	BoT-IoT, CICIoMT2024, simulation environments	IoT, IoMT, adaptive intelligent systems	Adaptation and sequential decision capability	Hyperparameter sensitivity and computational/training complexity
ML and ensemble learning	Botnet, network intrusion and multiclass attack detection	TON-IoT, N-BaIoT, WUSTL-IIoT-2021, Edge-IIoTset, RT_IoT2022	IoT and IIoT	Low inference overhead and strong performance on structured traffic	Dependence on feature representation and weaker rare-class detection
Explainable and adversarially robust AI	Interpretable detection and adversarial resilience	TON-IoT and adversarial benchmark datasets	IIoT and safety-critical intelligent systems	Improved transparency, forensic interpretation, and robustness assessment	Additional computational overhead and limited IIoT-specific adversarial validation

## 3 Cyber threats in IIoT and AI-driven mitigations

### 3.1 Threat classification in IIoT

Attacks on IIoT systems affect the systems at multiple architectural levels with distinct attack techniques and vulnerabilities. A tiered classification of threats facilitates the deployment of targeted AI-driven detection systems, enabling a comprehensive understanding of the impacts of cyberattacks on IIoT operations. The IIoT architecture comprises three operational stages: the perception layer, the network layer, and the application layer. Adversaries target weaknesses inherent in each of these layers in increasingly sophisticated ways.

#### 3.1.1 Perception layer threats

In AI-oriented IIoT security, the perception layer is the primary interface between the physical and digital worlds and forms the foundation of the IIoT architecture. It consists of embedded devices, an Radio-Frequency Identification (RFID) module, sensors, and actuators that continuously collect and transmit industrial data in real time. This layer is easily vulnerable to adversaries because these components are often low-power and resource-constrained and are commonly used in open environments. Compared with common IT environments, perception-layer IIoT devices lack robust OS-level security and advanced firmware protection. In smart factories, power grids, or oil refineries, attackers need basic tools to physically tamper with a sensor or modify its signal to gain access to the digital pipeline without authorization (Jangra and Rana, 2024; Ansar et al., 2023; Babu and Pal, 2024).

- **Node capture:** This attack happens when someone physically steals a device or tries to change its logic or access passwords. Thereafter, attackers may decide to reprogram the device to carry out more attacks or use stolen identities. Since there are no records or clear signs of behavior, it is hard to detect. Unusual behavior patterns, such as sudden changes in configuration or unexplained data spikes, can be spotted with lightweight AI models like RF and KNN by learning normal performance profiles. Because they require little processing power, these models can be used on-site for real-time analysis (Kornyo et al., 2023; Illi et al., 2023).
- **Spoofing:** Spoofing attack is an attack where the assailant masquerades as a genuine sensor or actuator in an effort to inject fake data into the IIoT network. This may result in control systems being fooled into performing dangerous actions, particularly in accurate manufacturing or energy automation. AI-based models such as SVM and Autoencoders capable of modeling identity-based data (such as packet frequency and device signature) and identifying attempts at impersonation by identifying excursions from learned behavior have been promising to use (El-Rashidy et al., 2025; Savadatti et al., 2024).
- **Radio jamming:** Jamming can cause delays, loss of data, or complete disconnection of a device. In this attack, the attacker disrupts wireless communications between devices in the perception layer and the network through interference. Traditional systems depend on reactive detection. Recent studies have instead used unsupervised learning methods like Isolation Forest and one-class SVM. These methods analyze transmission timeouts, communication failure rates, and signal strength to identify jamming patterns without any labeled data (Mai et al., 2024; Ahuja et al., 2024).

#### 3.1.2 Network layer threats

The Network Layer ensures reliable and secure communication between smart sensors, edge devices, and cloud-based control systems in AI-enabled IIoT setups. However, the distributed nature of IIoT networks and the use of wireless communications make the network layer particularly vulnerable to identity-based and advanced routing attacks. These attacks often resemble normal traffic flow, which makes them especially dangerous in the context of AI-enhanced IIoT security. They can be difficult to detect with firewall-based systems or through a set of static rules (Jahangeer et al., 2023; Hussain et al., 2024; Wisdom et al., 2024).

- **Sinkhole attacks:** To attract traffic from nearby nodes, a compromised node in this attack falsely advertises the best routes. The attacker can change, drop, or inspect the packets after redirecting the data. Deep learning models such as CNN-LSTM and LSTM can detect time-series anomalies in packet flow, like sudden route changes or inconsistent latency. They perform well in this scenario. These algorithms have demonstrated strong accuracy in identifying sinkhole patterns in simulated IIoT environments when trained on communication flow data (Omar et al., 2024; Hasan et al., 2025).
- **Selective forwarding:** In this case, an attacker tends to target critical information, such as system controls or notifications. They purposely drop some packets while forwarding others. It is still challenging to detect these selective packet manipulations with threshold-based systems. AI models such as Random Forest, GRU, and One-Class SVM analyze packet delivery consistency and communication behavior over time. These models can be used to determine if packet loss is intentional or due to a faulty link (Omar and Soudan, 2023).
- **Sybil attacks:** A single node will need to establish multiple identities to change routing systems or to participate in illegal communication. Knowing the behavior of distribution and identification is often required to detect these activities. CNNs' hybrid models with GRU have been successful in detecting anomalies and forecasting node identity patterns in the case of the presence of multiple identities by a single entity. Research on the combination of blockchain and AI also identifies the possibility of identity verification in IIoT mesh networks (Gupta et al., 2023; Abu Munshar et al., 2024).
- **Hello flood attacks:** Attackers send strong “hello” messages to trick nodes into believing that they are close neighbors. This results in sub-optimal routing and energy loss. Adding RSSI (Received Signal Strength Indicator) and energy profiles enables simple machine learning classifiers, such as SVM and KNN, to detect unusual signal patterns. These models can distinguish between real and fake broadcasts (Sandhya et al., 2025; Patel and Singh, 2023).

#### 3.1.3 Application layer threats

The application layer in the IIoT is responsible for the interfaces with the cloud, data analysis, data presentation, and decision-making. It enables communication between IIoT systems and external applications like cloud-based AI engines, automation apps, dashboards, and remote-control interfaces. If compromised at this level, it could introduce threats to operational integrity, privacy, and safety as it acts as the command-and-control interface for industrial operations. Application-layer targeted software exploits can evade lower-layer security and cause persistent disruptions (Eyeleko and Feng, 2023; Priyadarshini et al., 2023).

- **Ransomware:** Ransomware is a significant threat at the application level to IIoT systems. It can also jam important information and halt industrial processes. It primarily targets cloud APIs, control software, and HMIs. Unlike conventional malware, ransomware frequently exhibits a latent propagation phase prior to encryption execution, making early-stage behavioral detection particularly challenging. AI tools, such as CNNs and LSTM/GRU, are becoming more adept at spotting unusual access, file modifications, and command sequences. Mixed and unusual-event-based models are effective at resisting attack, but it remains challenging to deploy them on edge devices in real time. In industrial environments with safety as a critical concern, explainable AI frameworks are growing in use to support decisions and validate alerts (Jeon et al., 2023).
- **Session hijacking:** An attacker hijacks a valid user session by stealing or guessing session tokens. This can lead to privilege escalation, remote command execution, and data theft. These models can be used to detect anomalies like token reuse, session duration, and login patterns by examining request headers, session time, and frequency. Such models, coupled with behavioral biometrics and two-factor authentication (2FA), have been shown to enhance robustness in secure IIoT environments (Yanmin et al., 2025; Aslan et al., 2023).
- **Malware infiltration:** The prevalence of malware in IIoT applications is increasing, primarily due to the existence of cloud interfaces, third-party APIs, and untrustworthy software updates. In contrast to traditional information technology systems, a significant number of IIoT devices lack sandboxing capabilities and antivirus software. Several studies have indicated that Convolutional Neural Networks trained on binary metadata, network traffic, or opcode sequences have demonstrated high accuracy in classifying known malware families. Hybrid models, such as CNN combined with Autoencoders, are currently being investigated to enable the detection of zero-day malware by recognizing anomalous features (Raghuraman et al., 2024; Rana et al., 2024).

### 3.2 Detection complexity: threat behavior with model intelligence

Attacks on IIoT systems vary in their characteristics and the level of limitations they impose on detection. A firewall may detect a simple spoofing attack, but an anomaly-based machine-learning algorithm can easily reveal it. On the other hand, a forwarding attack may evade logic-based systems and even simple machine-learning models due to its indistinguishable nature from normal packet drops. Therefore, the attack type that poses challenges to detection is not the attack itself, but the extent to which the model can identify and characterize limitations. Traditional IIoT intrusion detection systems were rule-based, followed by pattern-based machine learning approaches such as support vector machines and decision trees. The focus has now shifted to AI techniques that analyze temporal behavior, specifically GRU and LSTM. This evolution is not merely for enhancement but is a direct response to contemporary IIoT attacks that are dynamic, stealthy, and temporal in nature (Bellamkonda, 2024; Raza et al., 2024; Mekala et al., 2023; Ni and Li, 2024). Table 4 provides a comparative framework linking IIoT threat characteristics with suitable AI techniques and their corresponding deployment requirements, explainability, computational complexity, and practical deployment feasibility. The comparison indicates that model suitability is determined not only by detection capability but also by the behavioral complexity of the threat and the computational constraints of the target IIoT environment.

**Table 4:** Comparative framework for threat complexity, AI model suitability, and deployment feasibility in IIoT (Bellamkonda, 2024; Omar et al., 2024; Gupta et al., 2023; Jeon et al., 2023).

Tier	Threat characteristics	Representative threats	Suitable AI techniques	Deployment requirements	Explainability	Computational complexity	Practical deployment feasibility
I	High-visibility, relatively distinct traffic or behavioral deviations	Spoofing, DoS, hello flood	RF, SVM, KNN	Lightweight feature extraction; limited compute and memory	Moderate–high^*^	Low	High—suitable for resource-constrained edge deployment
II	Behavioral and context-dependent attacks that may resemble legitimate activity	Sybil, selective forwarding	CNN, AE, CNN–GRU	Historical/ behavioral traffic features; moderate processing and memory resources	Low–moderate; *post-hoc* XAI may be required	Moderate	Moderate—more suitable for capable edge/fog nodes
III	Stealthy, sequential, multi-stage, or evolving attack behavior	Session hijacking, APT (advance persistent threat), zero-day	LSTM, GRU, transformers	Sequential/context-rich data; greater compute, memory, and inference capacity	Low without dedicated XAI mechanisms	High	Low–moderate—generally more suitable for fog/cloud or accelerated edge platforms

The qualitative ratings are synthesized from the representative studies reviewed in this section and are intended for relative comparison. Explainability reflects intrinsic interpretability and reliance on post-hoc XAI methods. Computational Complexity reflects relative training and inference requirements. Practical Deployment Feasibility reflects suitability for resource-constrained IIoT deployment considering computational, memory, and latency requirements. The asterisk (*) Indicates that the explainability rating may depend on the use of post-hoc XAI methods.

AI-driven intrusion detection approaches can conceptually be divided into three tiers, depending on the complexity of behavior and the intelligence needed to detect the various types of cyber threats. Tier I consist of lightweight, signature-based, and shallow machine learning methods that are designed to detect high-visibility attacks with clear behavioral characteristics. Tier II includes models based on knowledge of the context that can detect behavior and spatial attack patterns that are similar to normal network traffic. Tier III includes temporal intelligence models that can detect the sequential, stealthy, and multi-stage attack behaviors by the use of long-term contextual analysis. This is a conceptual hierarchical model to understand the relationships between threat complexity and AI model capacity, not an architectural classification.

**Tier I: Signature & threshold models—for shallow behavior**

Simple machine learning classifiers like Random Forest or SVM, and even signature-based detection rules, can be used to detect attacks like spoofing, DoS attacks, and hello floods. Such risks cause rapid traffic increases or unusual traffic patterns. In some instances, the accuracy of AI models can exceed 95% when they are trained using simple network features such as packet rate, node ID, and hop count. However, such models tend to produce numerous false alarms and are challenging to learn when the difference between true and false is small. They perform poorly in unstable industrial traffic conditions where the traffic does not typically follow a trend. These models are best suited for quick edge attack threats.

**Tier II: Context-aware learning—for behavioral attacks**

These threats require both behavioral and spatial modeling and are typically tackled by CNNs, autoencoders, or hybrid CNNs and GRU models. These models learn communication patterns between several nodes and are able to detect misbehavior based on node history and correlations among traffic patterns. But they suffer from data imbalance, require large training samples, and have poor performance in topologies with rapid changes, like mobile sensor networks or high-frequency industrial control systems. Shallow classifiers fail to perform well when attacks are similar to normal operations. Examples include:

- Selective forwarding: An attacker forwards some packets but drops critical ones.
- Sybil attacks: A single device acts as many devices using multiple identities.

**Tier III: Temporal intelligence—for stealthy, multi-stage attacks**

Eventually, complex attacks can emerge, such as session hijacking, malware infiltration, and zero-day exploits, that may emerge as a result of cloud vulnerabilities, slow system response, or weak identity verification. It takes time and context to recognize them. Transformer-based models, RNNs, LSTMs, and GRUs are relevant in this regard. By analyzing log sequences, API calls, or traffic patterns, they can detect “slow-drip” anomalies that rule-based systems fail to catch. Proper training can enable them to identify attacks that basic intrusion detection systems (IDSs) fail to detect. However:

- They need labeled time-series datasets.
- Their inference latency is too high for real-time edge deployment.
- They often lack explainability.

#### 3.2.1 Computational complexity and accuracy-efficiency trade-offs of AI architectures

In the context of Internet of Things (IoT) and Industrial Internet of Things (IIoT) intrusion detection, detection accuracy alone does not determine the suitability of an artificial intelligence (AI) model. Edge devices, gateways, and embedded healthcare systems are often resource-constrained and have strict requirements on computational complexity, memory usage, inference latency, and energy efficiency. Therefore, the choice of learning architecture must consider both predictive performance and computational cost, rather than focusing solely on benchmark accuracy. While architectures like transformers and graph neural networks are effective in representing complex attack patterns, they have significantly greater computational demands than traditional machine learning methods. In contrast, ensemble learning approaches such as Random Forest and XGBoost typically require less computational overhead and compete with other approaches on benchmark datasets. Table 5 presents the asymptotic computational complexity for some illustrative AI architectures discussed in this paper.

**Table 5:** Asymptotic computational complexity of representative AI architectures for IoT and IIoT intrusion detection.

Architecture	Approximate training complexity	Approximate inference complexity (per sample)	Dominant scaling factor
Support vector machine (RBF Kernel)	*O*(*n*^2^) − *O*(*n*^3^)	*O*(*n*_*sv*_*d*)	Number of training samples (*n*) and support vectors (*n*_*sv*_)
Random forest	*O*(*Tnd* log *n*)	*O*(*Td*)	Number of trees (*T*) and feature dimension (*d*)
XGBoost	*O*(*Tnd* log *n*)	*O*(*Td*)	Number of boosting trees (*T*) and feature dimension (*d*)
1D Convolutional neural network (CNN)	≈ *O*(*nLkC*_*in*_ *C*_*out*_)	≈ *O*(*LkC*_*in*_ *C*_*out*_)	Number of convolutional layers, kernels, and channels
LSTM/GRU	*O*(*th*^2^)	*O*(*th*^2^)	Sequence length (*t*) and hidden-state dimension (*h*)
Transformer (self-attention)	*O*(*t*^2^*d*)	*O*(*t*^2^*d*)	Quadratic dependence on sequence length (*t*)
Graph neural network (GNN)	*O*(*Ed* + *Vd*^2^)	*O*(*Ed* + *Vd*^2^)	Number of graph edges (E), graph nodes (V), and feature dimension (d)
Federated learning (FedAvg)	*O*(*K* × *LocalModelCost* + *CommunicationOverhead*)	Same as underlying local model	Number of participating clients (*K*) and communication rounds

The asymptotic computational complexities are synthesized from standard algorithmic analyses and the representative AI architectures discussed in this section.

Table 5 shows that various AI architectures possess distinct computational characteristics that directly influence their suitability for deployment in IoT and IIoT. The models based on transformers offer excellent long-range dependency modeling capabilities but are quadratic in terms of sequence length and not suitable for large-scale edge deployment. Likewise, Graph Neural Networks (GNNs) can also learn the structure of the device network and its interconnections, but they are computationally expensive and have been shown to increase in computational complexity as a function of the size and connectivity of the graph. Tree-based ensemble methods, however, typically offer a better performance/complexity trade-off, and they are appealing for use in intrusion detection in resource-limited applications. These observations confirm that there is an inherent Pareto frontier between accuracy and efficiency for these architectures in that no single one can achieve both optimal accuracy and minimal computational cost. Therefore, the choice of architecture must be based on the application requirements, deployment constraints, and available computational resources and not just on the accuracy of the benchmark.

### 3.3 Challenges in cyber threat detection for IIoT

Artificial intelligence's role in the cybersecurity of IIoT environments has proven useful in creating more sophisticated threat detection solutions. However, issues with operations and the environment have hindered the widespread adoption of these AI-driven solutions. The IIoT ecosystems consist of many different devices, applications that must operate in real-time with very low latency, and applications that are critical to the success of the business. These factors create significant disconnects between the potential of the algorithms and the performance of the devices in the field when compared against enterprise IT environments. Based on the review study, this section briefly summarizes the main challenges still to be overcome before deploying a system able to detect threats using AI in IIoT applications, as well as the challenges found in most of the studies (Alani, 2023; Pandey et al., 2024).

#### 3.3.1 Scarcity of labeled and high-quality data

One of the most fundamental barriers to training accurate AI models for threat detection is the lack of large-scale, labeled datasets; many IIoT environments are proprietary, and organizations are hesitant to share attack data due to privacy and regulatory concerns; and existing datasets, like NSL-KDD, CICIDS, and TON-IoT, are either outdated, have little industrial relevance, or are biased toward a specific network environments (Admass et al., 2024). As a result:

- Supervised ML/DL models suffer from underfitting or overfitting.
- Researchers resort to data augmentation, which may not represent real-world behavior.
- Transfer learning becomes less effective due to domain shift.

#### 3.3.2 Real-time processing constraints at the edge

IIoT systems have constrained processing power at the edge and strict latency windows. For both inference and retraining, AI models—particularly deep learning architectures like CNN with GRU or LSTM—frequently demand substantial computational resources. The majority of high-accuracy models are still made for fog/cloud environments, despite the emergence of edge AI accelerators and model reduction methods (such as pruning and quantization; Mohy-Eddine et al., 2023). Implications include the following:

- Difficulty in implementing real-time detection on systems with limited resources.
- High inference latency can result in missed detection of fast-acting threats.
- It is yet unclear how model depth and energy efficiency are traded off.

#### 3.3.3 High false positive (FP) and false negative (FN) rates

Particularly when training statistics are unbalanced or lack context variety, behavioral attacks—such as selective forwarding and sensor manipulation—can produce a high number of false positives because they closely resemble legal actions. On the other hand, because they deviate from the established threat patterns, covert APTs and zero-day vulnerabilities frequently produce false negatives. This erodes trust in the system, causing operators to:

- Ignore alerts (alert fatigue).
- Disable intrusion detection systems entirely.
- Misinterpret benign behavior as malicious.

#### 3.3.4 Concept drift and adaptive attacks

Industrial environments are dynamic. Network topologies vary, device responsibilities change, and traffic and behavior change with new firmware updates. The problem known as “concept drift” occurs when AI models that were trained on earlier data may no longer appropriately represent the current environment, which lowers detection. Moreover, adaptive attackers may develop their own AI models to explore and evade detection boundaries via adversarial machine learning. Such a continuous arms competition quickly makes static models obsolete. The solutions being investigated include:

- Continual learning and online training.
- Adversarial robust models and generative replay.
- Integration of self-healing detection pipelines.

#### 3.3.5 Lack of explainability in deep learning models

Deep learning has increased the accuracy of complex threat identification, but it also brings with it a significant problem: opacity. Decisions about security in industrial settings need to be open and auditable. However, it might be challenging to explain why a particular session or packet flow was detected because models like Transformers and LSTM frequently act like black boxes (Lone et al., 2023). This limits deployment in:

- Safety-critical systems (e.g., smart grid, oil & gas).
- Regulatory environments (e.g., GDPR, NIST compliance).

## 4 Datasets for AI-driven IoT and IIoT intrusion detection

### 4.1 Overview and selection criteria

The quality, diversity, and representativeness of benchmark datasets heavily determine the validity of machine learning or deep learning-based intrusion detection systems. The requirements of IoT and IIoT security datasets differ from traditional IT network datasets in several ways: First, the devices in IoT and IIoT networks are diverse, ranging from consumer, industrial, and medical, and are often connected to different protocols such as Message Queuing Telemetry Transport (MQTT), Modbus, Constrained Application Protocol (CoAP), and Zigbee; second, the natural class imbalance of IoT and IIoT security datasets is much more severe, with attack events occurring much less frequently in operational networks; and third, IoT and IIoT security datasets must capture not only network flow statistics but also device telemetry, system logs, and protocol-level payload information. These requirements have led to a rapid growth of purpose-built public IoT security datasets over the past 6 years. After 2021, the number of new datasets has grown rapidly as researchers have realized that existing IT benchmarks like NSL-KDD and CIC-IDS2017 are not suitable for IoT security evaluation (Kaur et al., 2023; De Keersmaeker et al., 2023).

This section summarizes 12 methodology-relevant, publicly available IoT and IIoT threat landscape datasets. It covers four application domains: general IoT botnets and network traffic, industrial IIoT testbeds, healthcare IoMT environments, and advanced persistent threat (APT) scenarios, and includes well-known benchmarks that have served as a foundation for much of the existing research, as well as the latest datasets published in 2025 and 2026. We explore data collection methods, network scope and devices involved, attack taxonomy, feature engineering, and the suitability of each dataset for machine learning, deep learning, and federated learning paradigms.

### 4.2 General IoT and IIoT network datasets

#### 4.2.1 Bot-IoT dataset

The Bot-IoT dataset was introduced by Koroniotis et al. (2019) and is still one of the most popular large-scale datasets for bot-IoT and network intrusion detection. The dataset was created in a simulated lab environment using Node-RED to simulate the behavior of IoT devices, such as smart refrigerators, weather stations, and lighting systems, and an AWS MQTT broker. Attack traffic was sent over the DDoS, DoS, reconnaissance, and data theft attacks on top of Transmission Control Protocol (TCP), User Datagram Protocol (UDP), and Hypertext Transfer Protocol (HTTP) protocols; network packets were captured using Tshark, and flow records were extracted using Argus. The resulting dataset includes more than 72 million labeled records, 30+ flow-based features, and 14 engineered statistical features, in both Packet Capture (PCAP) and Comma-Separated Values (CSV) formats, for both binary and multiclass classification use cases. Its large scale and attack diversity make it especially useful for testing deep learning architectures in realistic traffic loads. However, the dataset has limited use for forensics beyond the network layer, and the simulated device traffic may not represent real device traffic.

#### 4.2.2 TON-IoT telemetry dataset

In 2020, Alsaedi et al. (2020) introduced a new methodologically advanced dataset, TON-IoT, which combines three complementary data modalities: network traffic, operating system logs, and device telemetry, collected from seven different types of IoT/IIoT sensors such as Modbus sensors, GPS trackers, smart refrigerators, and thermostats. The data was gathered from a hybrid edge-fog-cloud testbed created with VMware NSX virtualization allowing for realistic simulation of industrial network architectures. It covers one of the most comprehensive attack taxonomies available in openly available IoT datasets, with nine attack categories represented, such as ransomware, XSS, DoS, DDoS, backdoor, injection, Man-in-the-Middle (MITM), scanning, and password cracking. Labeled ground truth data is stored in device-specific CSV files and system log formats (for both binary and multiclass classification). TON-IoT is a multi-modal dataset where flow, telemetry, and log data are all available at the same time, making it ideal for research on heterogeneous feature fusion. The main drawback is its computational complexity: the entire dataset requires extensive pre-processing and a lot of memory, making it difficult to benchmark the deployment as a lightweight application.

#### 4.2.3 WUSTL-IIoT-2021 dataset

Zolanvari et al. (2021) released the WUSTL-IIoT-2021 dataset in 2021. It contains 53 h of network traffic from a simulated industrial control environment, with only 7.28% of the records representing attack traffic. The class imbalance is realistic, unlike the common artificially balanced benchmarks, and is very useful for testing anomaly detection methods and studying model performance in the presence of false alarms, which are common in industrial applications. The data includes DoS, reconnaissance, command injection, and backdoor attacks, and 41 flow-level features are extracted using the Argus network monitoring tool, such as inter-packet timing, TCP round-trip time, jitter, and load statistics. The protocol coverage is limited to IP/TCP/UDP, and the attack taxonomy is more restricted than in more recent datasets, but its realistic traffic proportions and IIoT-specific flow metrics make it suitable as a complementary reference to test anomaly-based detection research, especially when assessing precision-recall trade-offs in natural imbalance.

#### 4.2.4 ACI-IoT-2023 dataset

Nack et al. (2024) at the Army Cyber Institute (ACI) created the ACI-IoT-2023 dataset, which includes 5 days of mixed-wired and wireless IoT network traffic from a realistic smart home testbed consisting of smart plugs, IP cameras, environmental sensors, and network switches. Port mirroring and tcpdump were used to capture the traffic, which was processed by CICFlowMeter-3.0 to extract more than 70 flow-based features in PCAP and CSV format. The attack scenarios are representative of the threat surface of consumer IoT environments: DoS, Address Resolution Protocol (ARP) spoofing, reconnaissance, and brute force attacks. The dual wired-wireless traffic capture, the diversity of devices in the dataset, and the granularity of flow-level features make it an attractive option for benchmarking intrusion detection in smart home scenarios. Some practical limitations are the lack of IPv6 traffic (which becomes more relevant as IoT device addressing evolves) and the size of raw PCAP files, which add storage and pre-processing overhead. However, its physical realism and flow-level granularity make it one of the more deployable benchmarks for network-layer IoT IDS evaluation.

#### 4.2.5 CICIoT2023 dataset

The CICIoT2023 dataset, released by Neto et al. (2023) at the Canadian Institute for Cybersecurity, is currently the largest real-world IoT security dataset, which is generated from a physical testbed of 105 heterogeneous IoT devices, such as Amazon Alexa, Google Nest, TP-Link smart plugs, Raspberry Pi boards, and Z-Wave/Zigbee sensors installed in a smart home environment. The network traffic was captured at line rate with a Gigamon TAP and Wireshark, resulting in 548 GB of raw PCAP data that was converted to 100 windowed flow and statistical features in labeled CSV format. The attack taxonomy includes 33 attack types grouped into seven categories: DDoS, DoS, reconnaissance, web-based, brute force, spoofing, and Mirai botnet variants, including attack propagation scenarios from IoT to IoT not modeled in previous datasets. The diversity of attacks covered and physical devices used makes CICIoT2023 the most ecologically valid general-purpose IoT IDS benchmark available today. The main research challenge is scale, as preprocessing or replicating the entire dataset demands significant computational resources and the number of attack classes is too large for minority classes, resulting in a severe class imbalance.

#### 4.2.6 X-IIoTID dataset

The X-IIoTID dataset was created by Al-Hawawreh et al. (2021) to address the connectivity and heterogeneity of devices in the context of the security assessment of IIoT. The dataset is comprised of 820,834 labeled instances, containing 67 features collected over 4 months on the Brown-IIoTbed testbed, which spans edge, cloud, and enterprise network tiers and includes both real and emulated devices, from which features were extracted from PCAP captures, Open Source Security Event Correlator (OSSEC) system logs, and SAR resource metrics. Represented are 18 advanced attack types, modeled with the Cyber Kill Chain (CKC), MITRE ATT&CK, and MALC frameworks, to ensure attack realism and coverage. The dataset meets 20 data quality requirements, which are not simultaneously satisfied by any previous public IIoT dataset and are therefore of particular interest for research in multi-view and multi-modal detection. The main drawback is that it does not have any predefined train-test splits, and it lacks cyber-physical process attack data, restricting its application to the purely network and log-based IDS paradigm.

### 4.3 Healthcare and medical IoT datasets

#### 4.3.1 IoT-healthcare dataset

The IoT-Healthcare dataset, created by Hussain et al. (2021) with the help of the IoT-Flock simulation framework, simulates the network traffic of a virtual intensive care unit (ICU) with 18 virtual medical devices, such as Electrocardiogram (ECG) monitors, glucometers, blood pressure sensors, and infusion pumps, communicating through MQTT and CoAP protocols. Normal operational traffic and cyberattack scenarios are captured, with network packets recorded by Wireshark and stored in PCAP and CSV formats, including application-layer payload information, and include attacks such as MQTT brute force, denial-of-service, SlowITE attacks, and more. The healthcare domain specificity of the dataset (protocol-level payload details, medical device communication patterns, and protocol stack awareness) makes it suitable for behavioral modeling and protocol-aware intrusion detection in healthcare environments. The main drawbacks of the dataset are that the device traffic is only simulated, there are no physical device behavioral signatures, and the attack coverage is relatively limited, all of which can potentially affect its direct applicability in real hospital network environments where there are a variety of physical devices and proprietary implementations of device protocols.

#### 4.3.2 CICIoMT2024 dataset

Dadkhah et al. (2024) published the CICIoMT2024 dataset at the Canadian Institute for Cybersecurity, the most extensive publicly available dataset for Internet of Medical Things (IoMT) security research. It was gathered from a testbed consisting of 40 IoMT devices, 25 physical and 15 simulated, communicating with each other using Wi-Fi, Bluetooth, and MQTT protocols, thus covering multiple protocols, as is found in real-world deployments of clinical IoT. This dataset contains 18 attack types captured from network taps with complete device behavioral profiling through idle, active, and connect/disconnect lifecycle states, ranging from DDoS, DoS, spoofing, and reconnaissance to MQTT-specific protocol attacks. This unique methodological contribution allows researchers to detect anomalies in device behavior patterns, not just flow-level statistics, enabling research on device fingerprinting and behavioral anomaly detection that flow-only datasets cannot support. The scale and multi-protocol nature of the dataset make processing challenging, particularly for lightweight or embedded IDS implementations; however, its ecological validity for healthcare IoT research surpasses that of other publicly available datasets.

### 4.4 Advanced and specialized IIoT datasets

#### 4.4.1 Edge-IIoTset dataset

Ferrag et al. (2022) proposed the Edge-IIoTset dataset, which is specifically tailored for both federated and centralized learning paradigms in the context of IDS research in contrast to datasets that are optimized for centralized model training only. It was gathered from a seven-layer hierarchical testbed that includes more than 10 physical sensors and technologies spanning edge, fog, cloud, blockchain, and SDN layers and various IoT communication protocols such as MQTT, Modbus, HTTP, and TCP. There are 14 attack categories (DoS, DDoS, MITM, injection, information gathering, and malware), and the features are selected from an initial set of 1,176 raw features in Zeek and TShark network analysis tools to a subset of 61 features for practical use. It is one of the most comprehensive multi-formats IIoT security datasets including PCAP captures, Zeek logs, system alerts, and resource metrics in CSV. Its explicit design for federated IDS benchmarking, which support both centralized and distributed model training, is particularly relevant for privacy-preserving IoT security research. The main deployment challenge is the feature dimensionality and complexity of the testbed, which can be too demanding for resource-constrained IoT nodes.

#### 4.4.2 CICAPT-IIoT dataset

Nearly all existing public IIoT security datasets lack advanced persistent threat (APT) scenarios, which is one of the most significant gaps in the current IIoT security dataset landscape, addressed by the CICAPT-IIoT dataset released by Ghiasvand et al. (2024) at the Canadian Institute for Cybersecurity in 2024. The dataset was generated over 168 h, including a semi-controlled hybrid testbed with real industrial devices (such as PLCs and smart cameras) and network infrastructure simulated with NS3, as well as provenance collection using SPADE, to model more than 20 APT attack techniques that span the data exfiltration, command-and-control, and lateral movement phases of APT29-style campaigns. Data is captured as PCAP and CSV flow files with 33 features and provenance graphs representing causal relationships among system events over longer time periods. The most unique technical aspect of the dataset is the provenance graph representation, which allows behavioral analysis across hours or days in multi-stage attack sequences that are not possible with flow-based datasets. The dataset's natural class imbalance (around 99.5% benign records) realistically reflects the stealthy, low-volume nature of APT activity, but it also poses a fundamental challenge for supervised learning methods that rely on a large number of labeled attack instances for training a model.

### 4.5 Emerging datasets: 2025–2026

#### 4.5.1 CIC-YNU-IoTMal 2026

The CIC-YNU-IoTMal 2026 dataset is one of the latest datasets released in the IoT malware detection benchmark area; it was created in collaboration with the Canadian Institute for Cybersecurity and Yunnan University (Dadkhah et al., 2026). It is tailored to tackle the increasing propagation of IoT malware by recording the malicious behavior of binary execution, network communication, and device-level telemetry on a wide variety of physical IoT devices when infected and subjected to controlled conditions. The dataset also adds fine-grained malware family labeling, allowing for multiclass malware classification instead of binary benign/malicious classification, and is gathered in a manner that minimizes the simulator-to-physical-device gap that exists in previous emulation-based sets and reduces the realism of such sets. The 2026 release makes it useful for research on the growing IoT malware landscape, such as botnets recruiting from a wide variety of devices and exploits targeting the firmware. This is a relatively new dataset, and there are few large-scale comparisons against existing baselines in the published literature, which is another opportunity for original contribution.

#### 4.5.2 DataSense IIoT dataset 2025

The DataSense IIoT dataset was created specifically for the IIoT security research community and is suitable for operational technology (OT) security environments and cyber-physical system (CPS) attack scenarios (Firouzi et al., 2025). Unlike existing IIoT datasets that only include network-layer traffic, DataSense IIoT combines sensor telemetry, process variable measurements, and network flow data from a live industrial testbed that includes SCADA components, PLC-controlled actuators, industrial Ethernet protocols such as PROFINET and EtherNet/IP, and standard TCP/IP traffic. The multi-layer data collection across both the IT and OT network tiers enables research into cross-domain attack propagation, a threat vector growing in importance as industrial networks merge with enterprise IT networks. The dataset contains attack scenarios that cover the cyber-physical threat spectrum, which is not included in any previous public IIoT dataset, such as process manipulation attacks, sensor spoofing, attacks on industrial control protocols, and conventional network intrusions. Its multi-layer feature set enables both network-centric and process-aware anomaly detection methods, making it especially relevant for assessing next-generation AI-powered ICS and SCADA security solutions for 2025.

### 4.6 Comparative analysis

**Figure 3:**
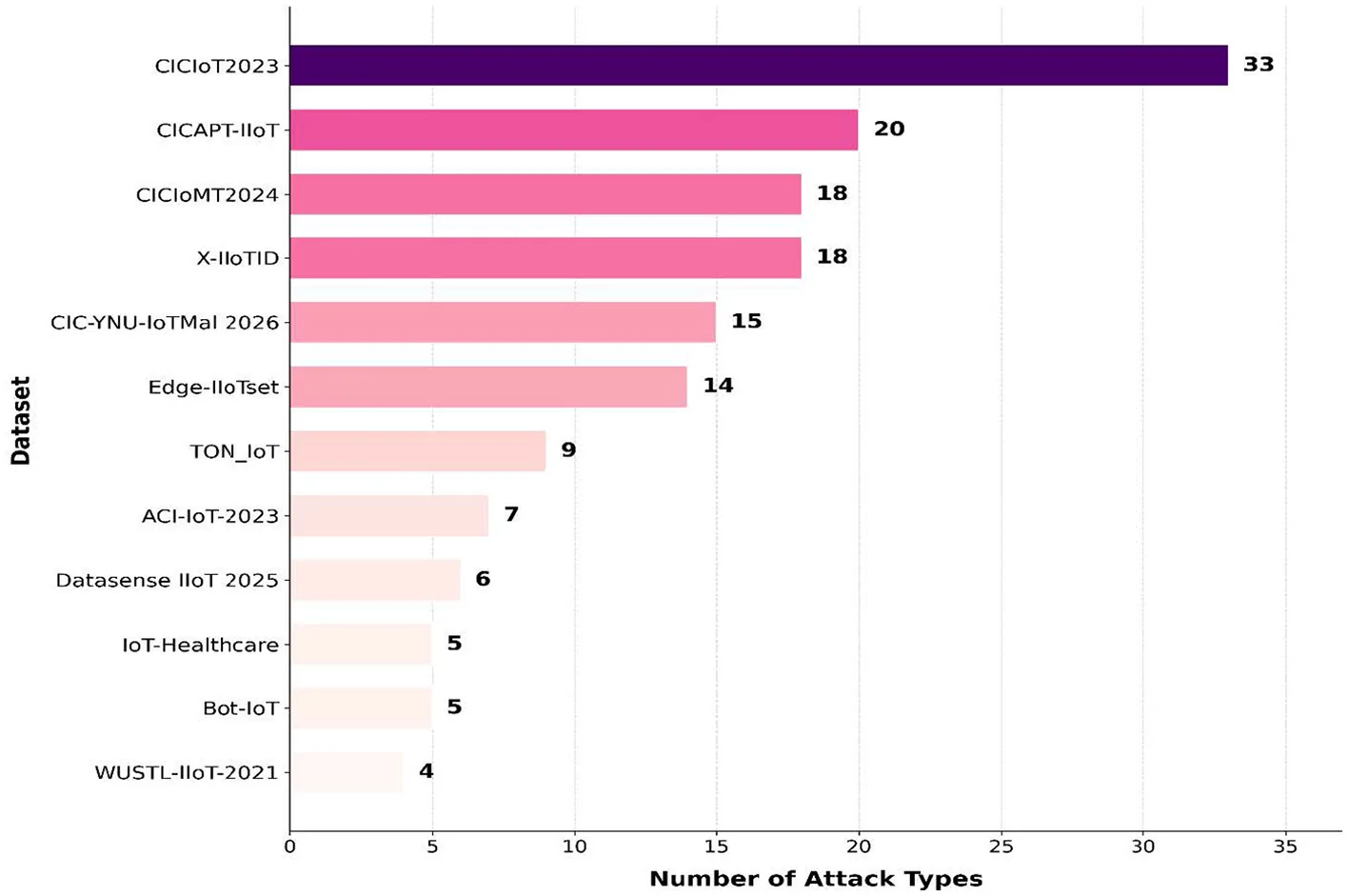
Attack type coverage per dataset.

The 12 datasets reviewed in this section collectively reveal several important trends and trade-offs in IoT and IIoT security dataset design. Figure 3 presents attack type coverage across datasets: CICIoT2023 leads with 33 distinct attack types, making it the most suitable benchmark for multiclass intrusion classification, while CICAPT-IIoT and X-IIoTID provide the deepest coverage of advanced and multi-stage threats. Datasets such as WUSTL-IIoT-2021 and ACI-IoT-2023, with narrower attack taxonomies, are better suited to focused DoS and reconnaissance detection scenarios where depth of coverage matters more than breadth.

**Figure 4:**
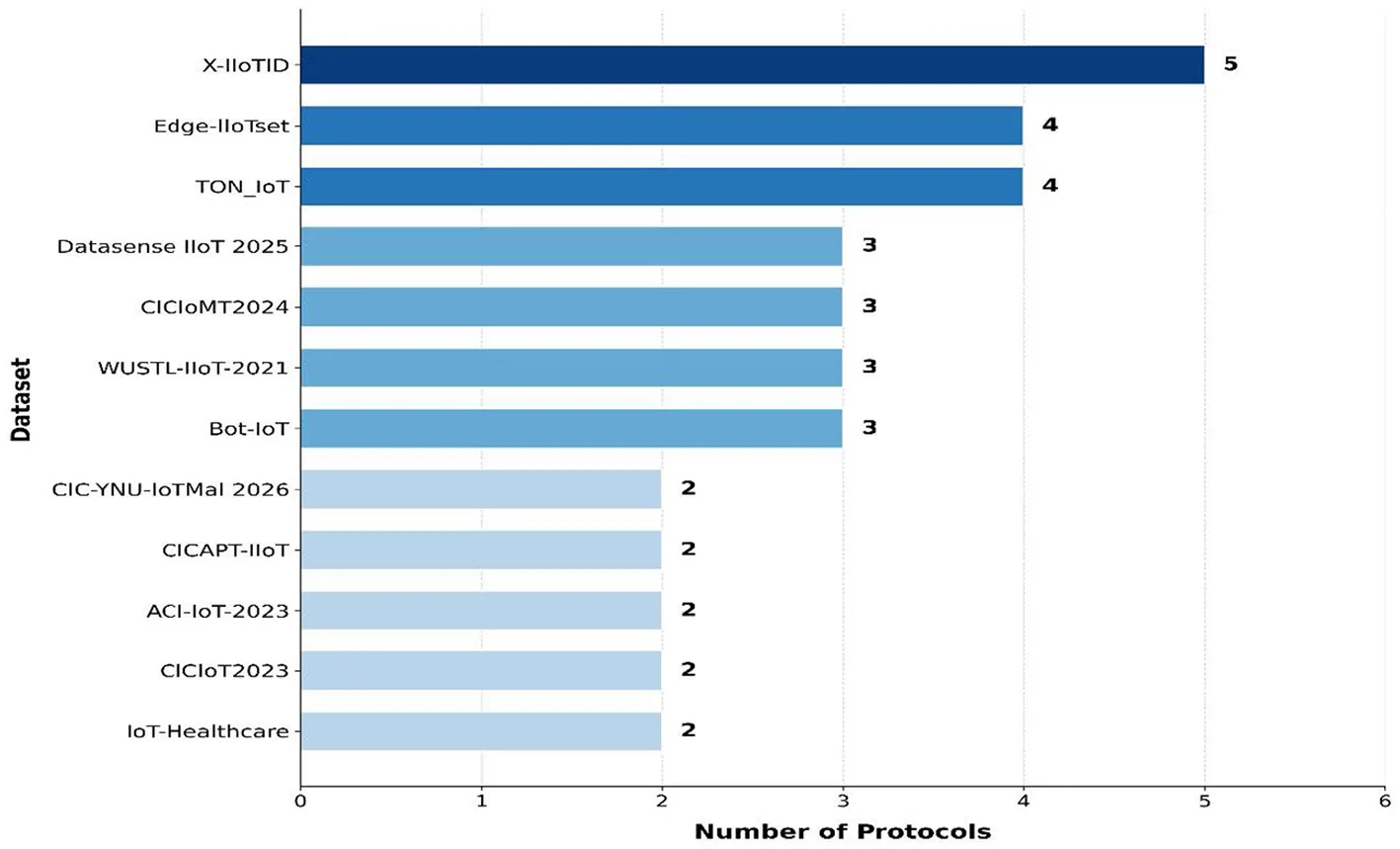
Diversity of protocols per dataset.

**Figure 5:**
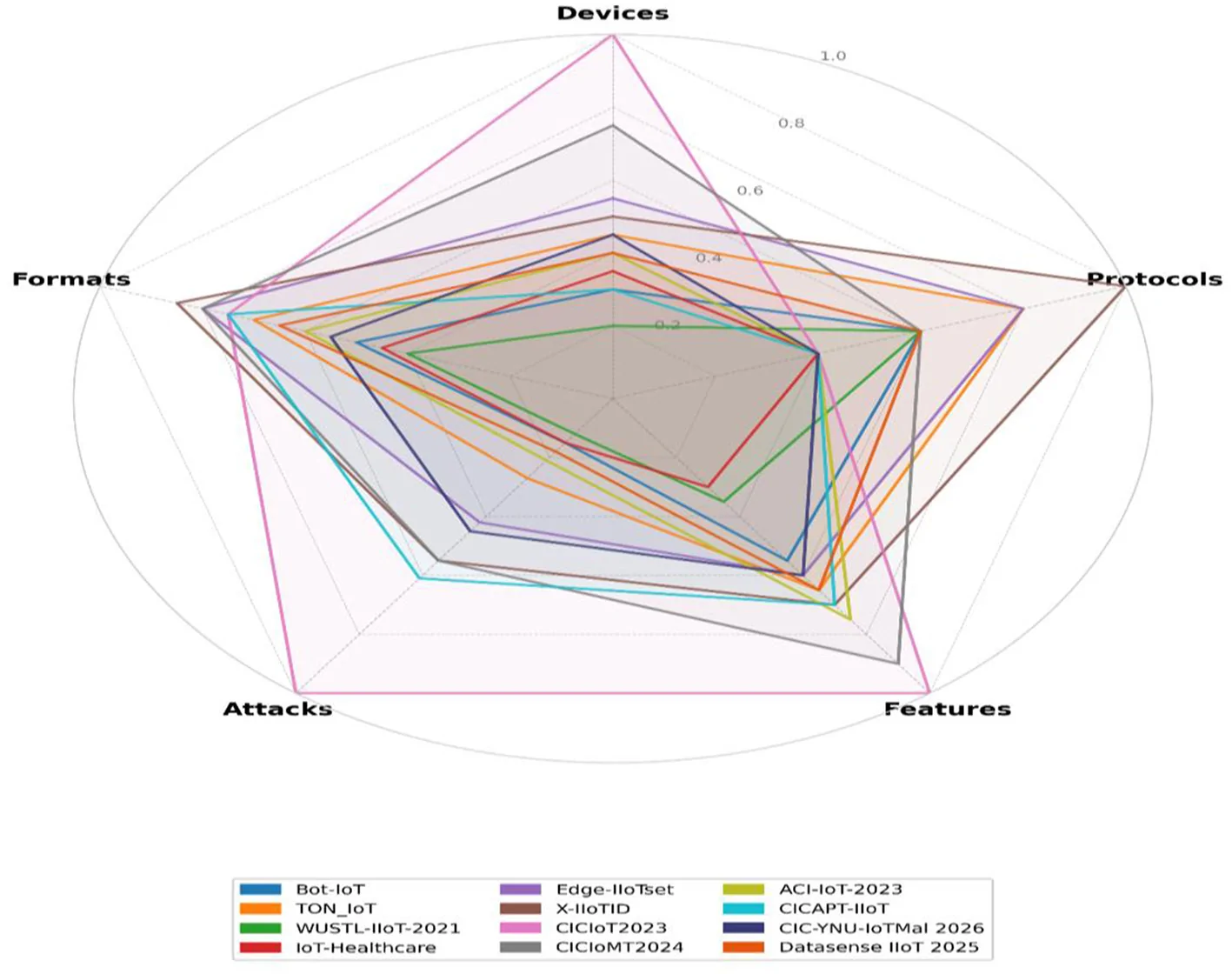
Features richness across datasets.

Figure 4 depicts protocol diversity, a crucial factor for real-world applicability. X-IIoTID and Edge-IIoTset support the widest protocol stack, up to five different protocols such as MQTT, Modbus, CoAP, TCP, and SMTP, which are the most representative of real IIoT heterogeneity. Datasets restricted to TCP/IP traffic, however, are less representative of a given protocol's attack surface and are typically less complex in terms of calculations. The radar chart in Figure 5 evaluates feature richness across five dimensions—device count, protocol support, feature count, attack diversity, and data format variety. CICIoT2023 and ACI-IoT-2023 demonstrate comparatively strong multidimensional characteristics across the evaluated feature dimensions, whereas WUSTL-IIoT-2021 and IoT-Healthcare exhibit comparatively narrower protocol and device diversity.

**Figure 6:**
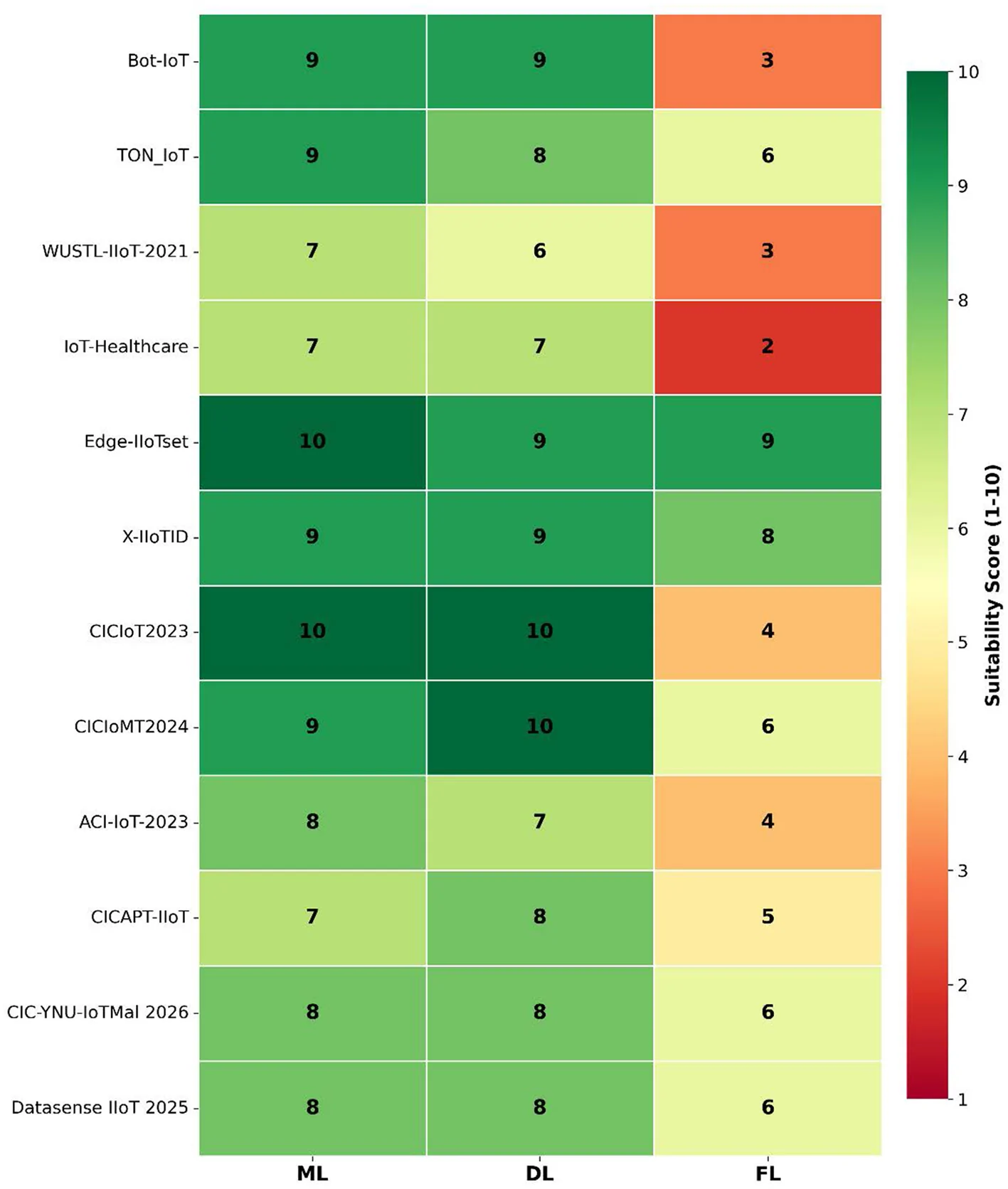
Dataset suitability score for machine learning, deep learning, and federated learning.

**Figure 7:**
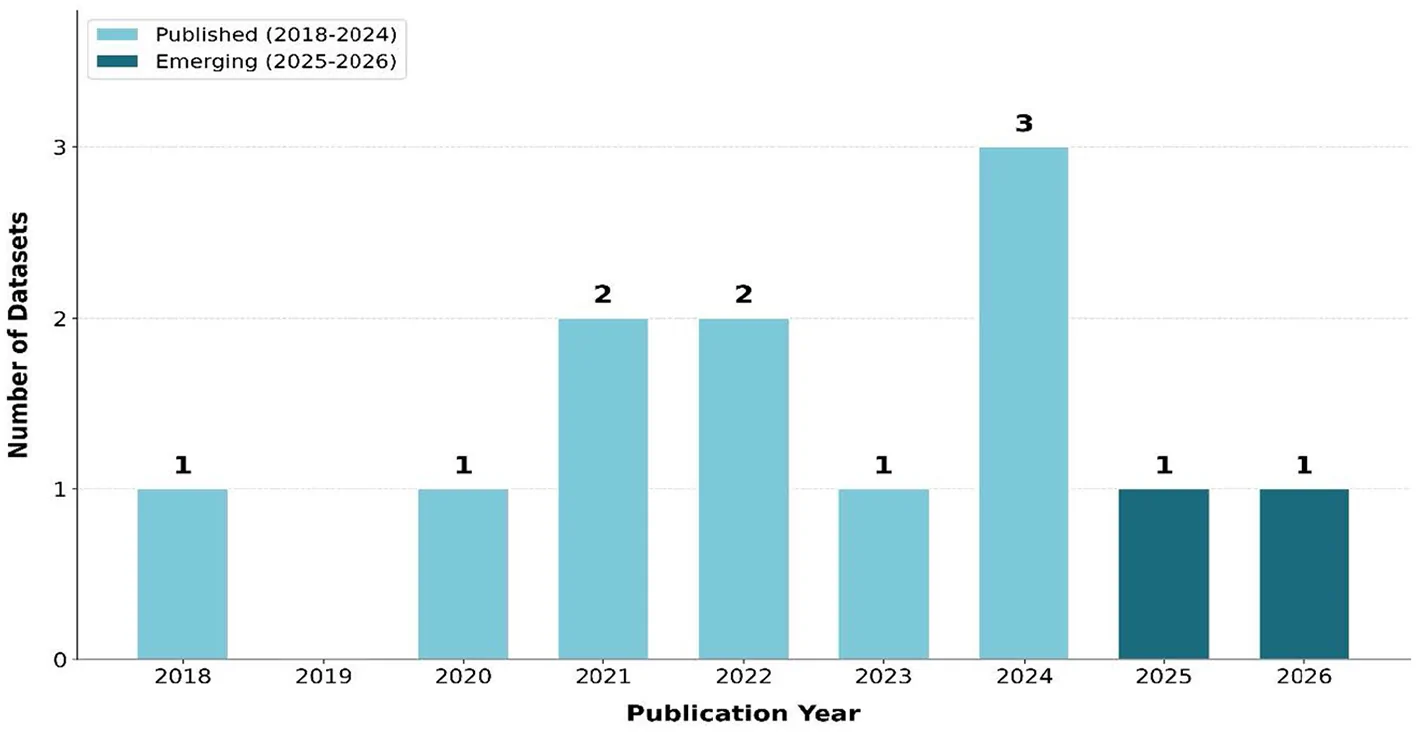
Dataset publication trend from 2018 to 2026.

Figure 6 provides a qualitative comparison of the relative suitability of each dataset for machine learning, deep learning, and federated learning based on dataset characteristics, including feature richness, data diversity, and support for distributed learning scenarios. The three paradigms that score highest are CICIoT2023, CICIoMT2024, and Edge-IIoTset, each offering a large labeled dataset, a rich feature set, and the ability to be distributed for training. Datasets like IoT-Healthcare, with centralized collection architectures, and Bot-IoT, which lacks metadata on devices, have lower applicability for federated learning. The publication trend of IIoT security datasets between 2018 and 2026 is illustrated in Figure 7, which shows that the number of datasets is increasing over the years and that 2023 is the year with the highest number of datasets.

Table 6 provides a consolidated comparison of all 12 datasets across their essential characteristics. Taken together, these datasets cover the primary axes of IoT security research from large-scale botnet detection and multiclass attack classification to healthcare-specific behavioral profiling, federated learning support, APT scenario modeling, and emerging cyber-physical threat coverage, but no single dataset satisfies all requirements simultaneously. The choice of benchmark must therefore be driven by the specific research question, threat model, and deployment context, with multi-dataset evaluation across complementary benchmarks representing the most robust validation strategy for generalizable IoT IDS development.

**Table 6:** Summary of public datasets in IIoT intrusion detection systems [Bot-IoT (Koroniotis et al., 2019), TON-IoT (Alsaedi et al., 2020), X-IIoTID (Al-Hawawreh et al., 2021), Edge-IIoTset (Ferrag et al., 2022), CICIoT2023 (Neto et al., 2023), and CICIoMT2024 (Dadkhah et al., 2024)].

Dataset	Device types	Protocols	Attack types	Collection method	Features	ML/DL/FL suitability	Key strength	Limitation
Bot-IoT	Simulated IoT devices including smart refrigerator, weather station, lighting systems	TCP, UDP, HTTP, MQTT	DDoS, DoS, reconnaissance, data theft (4+)	Simulated lab with node-RED and AWS MQTT broker; Tshark + Argus	30+ flow and 14 statistical features	ML, DL	Large-scale traffic and attack diversity	Simulated device behavior limits real-world deployment generalizability
TON-IoT	7 IoT/IIoT device categories including Modbus sensors and GPS trackers	MQTT, Modbus, TCP/IP	Ransomware, XSS, DoS, DDoS, backdoor, injection, MITM, scanning, password attacks (9)	Edge–fog–cloud VMware NSX testbed	Multi-modal telemetry, logs, and network features	ML, DL, FL	Multi-modal heterogeneous data	Heterogeneous telemetry requires complex feature integration and preprocessing
WUSTL-IIoT-2021	Simulated industrial control environment	TCP, UDP, IP	DoS, reconnaissance, command injection, backdoor (4)	53-h IIoT traffic capture using Argus	41 flow-level features	ML, DL	Realistic class imbalance	Limited protocol diversity restricts broader IIoT attack representation
ACI-IoT-2023	Smart plugs, IP cameras, environmental sensors, switches	Wired/Wireless TCP/IP	DoS, ARP spoofing, reconnaissance, brute force (4)	Smart-home testbed using tcpdump and CICFlowMeter	70+ flow features	ML, DL	Realistic smart-home deployment	Lacks IPv6 traffic and large-scale industrial deployment scenarios
CICIoT2023	105 heterogeneous physical IoT devices	Wi-Fi, Zigbee, Z-Wave, TCP/IP	33 attacks across 7 categories	Physical smart-home environment using Gigamon TAP and Wireshark	100 statistical and flow features	ML, DL, FL	Largest ecologically valid IoT dataset	Large data volume and severe class imbalance increase preprocessing complexity
X-IIoTID	Real and emulated IIoT devices across edge-cloud-enterprise layers	TCP/IP, enterprise protocols	18 advanced attacks	Brown-IIoTbed with PCAP, OSSEC, and SAR monitoring	67 multi-view features	ML, DL	CKC and MITRE ATT&CK alignment	Does not model cyber-physical process attacks or physical consequences
IoT-healthcare	18 virtual medical devices	MQTT, CoAP	MQTT brute force, DoS, SlowITE, protocol attacks	IoT-Flock ICU simulation environment	Network and payload-level features	ML, DL	Healthcare-specific traffic behavior	Fully simulated environment limits clinical deployment realism
CICIoMT2024	40 IoMT devices (25 physical, 15 simulated)	Wi-Fi, Bluetooth, MQTT	18 attack types	Multi-protocol IoMT clinical testbed	Behavioral and lifecycle-aware features	ML, DL, FL	Most comprehensive IoMT benchmark	Temporal behavioral profiling increases feature extraction and preprocessing complexity
Edge-IIoTset	Multi-layer edge-fog-cloud IIoT environment	MQTT, Modbus, HTTP, TCP	DoS, DDoS, MITM, injection, malware, information gathering (14)	Seven-layer IIoT architecture using Zeek and TShark	61 selected features from 1,176 raw attributes	ML, DL, FL	Explicit federated learning suitability	High-dimensional features require careful feature selection before model training
CICAPT-IIoT	PLCs, smart cameras, simulated industrial devices	TCP/IP, industrial traffic	20+ APT techniques	Hybrid industrial environment with SPADE provenance graphs	33 flow and provenance features	DL	Multi-stage APT behavioral analysis	Extreme class imbalance challenges robust APT detection performance
CIC-YNU-IoTMal	Diverse physical IoT devices infected with malware	Device-specific network protocols	Multi-family IoT malware behaviors	Controlled malware execution environment	Static and dynamic malware behavior features	ML, DL	Fine-grained malware family labeling	Limited benchmarking against existing malware detection datasets
DataSense IIoT	SCADA devices, PLCs, industrial actuators	PROFINET, EtherNet/IP, TCP/IP	Process manipulation, spoofing, industrial protocol attacks	Live OT/ICS industrial testbed	Cross-domain IT–OT features	ML, DL, FL	Cyber-physical process awareness	Multi-layer data synchronization increases preprocessing and deployment complexity

#### 4.6.1 Computational complexity and dataset scalability

The computational properties of benchmark datasets are as important as their feature coverage, protocol coverage, and attack diversity. The vast amount of raw network traffic and the high dimensionality of feature representations in large-scale datasets (like CICIoT2023 and CICIoMT2024) demand massive amounts of storage space, preprocessing time, and memory usage due to data collected from multiple protocols. In addition, multi-modal datasets, like TON-IoT and Edge-IIoTset, add to the computational overhead, as the collected telemetry, operating system logs, and network flow data need to be combined prior to model training. WUSTL-IIoT-2021 and ACI-IoT-2023, on the other hand, have comparatively smaller feature sets and simpler protocol coverage, which makes them more suitable for resource-constrained computing environments, but with comparatively limited attack diversity. A summary of the computational characteristics of the datasets reviewed is provided in Table 7 and includes a qualitative evaluation of raw data size, feature characteristics, computational preprocessing requirements, and overall computational burden.

**Table 7:** Computational characteristics of public IoT and IIoT datasets (Alsaedi et al., 2020; Al-Hawawreh et al., 2021; Ferrag et al., 2022; Neto et al., 2023; Dadkhah et al., 2024).

Dataset	Raw data scale	Feature complexity	Preprocessing complexity	Overall computational burden
Bot-IoT	Very large	Moderate	Moderate	Moderate
TON-IoT	Large	Multi-modal	High	High
WUSTL-IIoT-2021	Moderate	Low	Low	Low
ACI-IoT-2023	Moderate	Moderate	Moderate	Moderate
CICIoT2023	Extremely large (548 GB PCAP)	High	Very high	Very high
X-IIoTID	Large	High	High	High
IoT-Healthcare	Moderate	Moderate	Moderate	Moderate
CICIoMT2024	Large	High	High	High
Edge-IIoTset	Large	Very high (1,176 → 61 features)	High	High
CICAPT-IIoT	Moderate	High	High	High
CIC-YNU-IoTMal 2026	Large	High	High	High
DataSense IIoT 2025	Large	Multi-layer IT–OT	High	High

No existing dataset is comprehensive enough to cover both attack diversity and protocol coverage while also being realistic in terms of deployment and efficient in terms of computation, whether considered individually or in combination. Neither a single dataset nor multiple datasets exist that achieve maximum attack diversity and protocol coverage and are realistic and efficient in terms of computation, as is shown in Table 7. As a general rule, the more features a dataset represents and the more attack categories it covers, the more time, space, and memory are required to process, store, and hold the data, while lightweight datasets will have a more limited set of attack categories but will support a narrower set of protocols. These observations suggest a fundamental trade-off in the relationship between the richness of the data sets and their computational efficiency, and the selection of the benchmarks should consider both computational feasibility and deployment goals and experimental needs and should not be based exclusively on benchmark size or diversity of attacks. Together, Table 7 and Figure 6 provide complementary perspectives by evaluating both dataset suitability for AI paradigms and the associated computational burden, thereby enabling more informed benchmark selection for AI-driven IoT and IIoT intrusion detection research.

#### 4.6.2 Geographic bias and generalizability of public IIoT datasets

A key restriction of existing public IoT and IIoT intrusion datasets is the high level of geographic concentration. Major benchmark datasets are mostly from select research labs in Australia (Bot-IoT, TON-IoT), Canada (CIC-IDS series, CICIoT2023, and CICIoMT2024), and the United States (WUSTL-IIoT-2021 and ACI-IoT-2023). Although these datasets have greatly contributed to the development of AI-based intrusion detection research by offering a set of standard evaluation benchmarks, they only cover part of the industrial world.

Due to differences in manufacturing techniques, industrial standards, legacy equipment, and vendor ecosystems, industrial communication infrastructures differ among geographical regions. As a result, protocols are region-specific. For instance, PROFINET and PROFIBUS are extensively used in European manufacturing, Modbus is used by many industrial control systems in North America, and CC-Link is used in many industrial control systems in Japan. Likewise, different industries and national infrastructures have a different success rate in the implementation of EtherNet/IP, OPC UA, DNP3, and IEC 61850. However, the current public benchmark datasets are heavily skewed toward Modbus-, MQTT-, and traditional IP-based traffic and offer relatively few samples of a number of region-specific industrial protocols.

This geographical bias reduces the external validity and generalizability of intrusion detection models based on AI. Models that are trained on geographically localized data can learn protocol-specific communication patterns and vendor-specific traffic characteristics, rather than attack behaviors that are representative of the entire industrial environments. As a result, models that achieve high benchmark scores may exhibit lower detection accuracy, more prone to false alarms, and less robust when deployed in environments with other protocols, network architectures, and operating styles.

Industrial communication protocols such as PROFIBUS, PROFINET, Modbus, EtherNet/IP, OPC UA, DNP3, IEC 61850, and CC-Link should therefore be included in the future in representative implementations in geographically diverse industrial environments in IIoT benchmark datasets. A geographically representative dataset would enhance the external validity of research in intrusion detection using AI and aid in creating models that could be effectively deployed in various global IIoT settings.

### 4.7 Dataset selection framework and generalization considerations

While recent IoT and IIoT security datasets have broadened in scale, attack diversity, and protocol coverage, it is important to consider benchmark performance and dataset characteristics together. There is a wide range of realism in datasets, with some publicly available benchmarks using simulated or controlled testbed environments that do not necessarily reflect real-world deployment scenarios, including the variety of devices, protocols, and background traffic. As a result, models developed using these datasets are likely to perform well on the same benchmark but may suffer performance loss in other operational settings. Further, the presence of benchmark bias is also a major concern, as a few datasets are used repeatedly for their performance assessment, and the apparent improvement may be a result of adaptation to the characteristics of that benchmark. Additionally, differences in devices, communication protocols, traffic distribution, attack implementation, and feature representation result in domain shift, which further hinders cross-dataset generalization. Furthermore, the highly class imbalanced nature of many IoT and IIoT datasets can result in misleading accuracy scores and fail to reveal poor detection rates for attack classes that are not dominant. Thus, the choice of a dataset should not only be based on the size and diversity of attacks in the dataset but also on the realistic deployment, the coverage of the protocols, the class distribution of the attacks, and the intended operational environment.

Datasets should be selected according to the research objective and not for the purpose of benchmarking popularity, as the aim is to improve the reproducible and application-driven evaluation of the datasets. Table 8 provides a decision-centric overview of representative IoT and IIoT datasets, mapping and listing the major benefits and practical limitations for each in the context of common research scenarios.

**Table 8:** Dataset selection framework for AI-driven IoT and IIoT intrusion detection systems.

Research objective	Recommended dataset(s)	Selection rationale	Principal limitation
Large-scale multiclass intrusion detection	CICIoT2023	Extensive attack diversity and large-scale heterogeneous IoT traffic	Severe class imbalance and high computational requirements
Industrial IIoT intrusion detection	TON-IoT, X-IIoTID, Edge-IIoTset	Industrial architectures, heterogeneous protocols, and multi-modal data	Testbed-specific distributions may limit deployment generalization
Healthcare IoMT intrusion detection	CICIoMT2024, IoT-Healthcare	Medical-device communication and healthcare-specific attack scenarios	IoT-Healthcare relies on simulated devices; CICIoMT2024 requires substantial computational resources
Federated learning research	Edge-IIoTset, TON-IoT, CICIoMT2024	Support heterogeneous and distributed learning environments	Realistic non-IID partitioning remains challenging
Resource-constrained edge IDS	WUSTL-IIoT-2021, ACI-IoT-2023	Moderate feature sets and manageable computational requirements	Limited protocol and attack diversity
Advanced persistent threat detection	CICAPT-IIoT, X-IIoTID	Multi-stage attack representation and advanced threat modeling	Extreme class imbalance and specialized attack scope
Malware detection	CIC-YNU-IoTMal	Malware-family behavioral analysis	Limited comparative studies because of recent release
Cyber-physical security	DataSense IIoT	Combined network, telemetry, and industrial process information	Higher preprocessing and deployment complexity
Cross-dataset generalization studies	Multiple complementary datasets	Assesses model robustness under heterogeneous traffic distributions, device types, and protocol environments.	Feature harmonization and label consistency required

Future studies should use consistent benchmarking to allow fair comparison between different AI-based intrusion detection models. The training, validation, and testing sets should not be mixed to avoid information leakage, and all preprocessing, normalization, feature selection, and data balancing must be done only on the training set. In cases of a highly imbalanced dataset, consider using precision, recall, F1-score, false-positive rate, MCC, or other class-sensitive metrics, in addition to overall accuracy for performance evaluation. When deployment feasibility, computational characteristics including inference time, the amount of memory required, and the complexity of the model should also be reported. Whenever multiple benchmark datasets are available, models should also be tested on a set of complementary benchmarks to ensure that they can generalize to other datasets and improve confidence in their deployment in the wild.

## 5 Performance evaluation metrics

Accuracy alone is not a satisfactory measure of evaluation in the case of AI-driven cyber threat detection in the IIoT. The severe class imbalance that characterizes IIoT intrusion datasets makes accuracy an unreliable performance metric. Although accuracy may appear deceptively high, it can also make a model appear stronger than it actually is, particularly in the case of threats like selective forwarding or spoofing, which only take place in very low frequencies. Thus, precision, recall, F1-score, FPR, MCC, and AUC-ROC have become the conventional ways of quantifying security in IIoT studies (Aldhaheri et al., 2023; Swessi and Idoudi, 2022; Sivasankari and Kamalakkannan, 2022). The confusion matrix, which includes TP, FP, TN, and FN, yields these numbers. This matrix presents a clear image of the model's effectiveness in differentiating between normal and various forms of attack. The principal measures were the following:

- **True positive (TP):** When the system correctly identifies positive cases, they are called True Positives. This means correctly identifying and classifying situations as security threats in cybersecurity.
- **False negative (FN):** A false negative occurs when the system incorrectly marks an attack instance as benign when it is actually positive. This is what happens in cybersecurity when a system cannot find a real security threat.
- **False positive (FP):** False positives are cases in which the system incorrectly labels benign traffic as malicious when it is not. This scenario occurs when the system sets off an alarm for normal behavior.
- **True negative (TN):** When the system correctly identifies negative cases, they are called True Negatives. This type of detection means correctly identifying when normal behaviors are not a threat in cybersecurity.

Table 9 displays the connections among the aforementioned confusion matrix elements. Additionally, these elements can produce several evaluation metrics.

**Table 9:** Confusion matrix.

Confusion matrix	Predicted negative	Predicted positive
Actual negative	TN	FP
Actual positive	FN	TP

### 5.1 Accuracy

Accuracy measures the overall classification performance of a model by quantifying the proportion of correctly classified instances among all predictions. This metric provides a general idea of how well a system works. Equation 1 was used to determine this number.


$$\begin{array}{l} ACC=\;\left(TP\;+\;TN\right)\;/\;\left(TP\;+\;TN\;+\;FP\;+\;FN\right) \end{array}$$ (1)

### 5.2 Precision (positive predictive value)

Precision is the proportion of predicted attack instances the algorithm correctly predicts as positive. It measures how well a system can avoid false positives. Higher precision implies fewer false alarms. Equation 2 was used to determine this number.


$$\begin{array}{l} PR=\;TP\;/\;\left(TP\;+\;FP\right) \end{array}$$ (2)

### 5.3 Recall (sensitivity, true positive rate)

Recall is the percentage of the truly positive cases that the system can correctly recognize. It is a measure of the ability of a system to prevent false negatives. Higher recall indicates that a greater proportion of actual attacks are correctly detected. This score was calculated using Equation 3.


$$\begin{array}{l} R=\;TP\;/\;\left(TP\;+\;FN\right) \end{array}$$ (3)

### 5.4 F1-score

The F1-score is the harmonic mean of precision and recall. This provides a balanced assessment of how well a model works. It is calculated using Equation 4.


$$\begin{array}{l} F1=\frac{2\times Precision\times Recall}{Precision+Recall} \end{array}$$ (4)

### 5.5 False positive rate

The False Positive Rate (FPR), or fall-out rate, is the percentage of traffic records that the model incorrectly identifies as attacks when they are actually benign. It is computed as shown in Equation 5:


$$\begin{array}{l} FPR=\frac{FP}{FP+TN} \end{array}$$ (5)

### 5.6 Detection rate

The Detection Rate (DR), or Recall (True Positive Rate), is also mentioned as a separate metric in the IIoT security literature due to its close operational interpretation: the percentage of actual cyberattacks that the deployed IDS is able to detect before they are able to cause damage. It is computed identically to the recall as given in Equation 3.

### 5.7 Matthews correlation coefficient

The Matthews Correlation Coefficient (MCC) is a balanced evaluation measure that takes all four cells of the confusion matrix into account; thus, it is very useful for measuring classifiers on highly imbalanced datasets. In contrast to the F1-score, where only the positive class is taken into account, MCC is computed symmetrically for both classes and is equal to +1 if the classification is perfect, 0 if it is random, and −1 if the classification is completely inverse. It is computed as shown in Equation 6:


$$\begin{array}{l} MCC=\frac{TP\times TN-FP\times FN}{\sqrt{\left(TP+FP\right)\left(TP+FN\right)\left(TN+FP\right)\left(TN+FN\right)}} \end{array}$$ (6)

### 5.8 AUC-ROC

The Area Under the Receiver Operating Characteristic Curve (AUC-ROC) is the total discriminating power of a classification model over all possible decision thresholds, not just one fixed operating point. The ROC curve is a graph that shows the true positive rate (recall) against the false positive rate for each threshold value, and the area under the curve (AUC) is a scalar value between 0 and 1 that summarizes the curve. An AUC of 1.0 means perfect discrimination between attack and benign traffic at all threshold settings, and an AUC of 0.5 means there is no better-than-random discrimination. AUC-ROC is not tied to a specific threshold; thus, it is very useful for comparing models for different deployment settings with different optimal points to balance precision and recall, as well as for assessing the robustness of a model for different class distributions.

### 5.9 Critical comparison of evaluation metrics and deployment

The evaluation metrics discussed in Sections 5.1–5.8 are common in the domain of intrusion detection studies involving artificial intelligence, but they are not equally applicable across all evaluation datasets and deployment purposes. In IIoT cybersecurity, the dataset usually has a severe class imbalance, with much more benign traffic than cyberattack instances. In this case, accuracy alone cannot reliably measure classifier performance, since a classifier biased toward the majority class can have high accuracy but not detect minority attack classes. As such, the choice of evaluation metrics should be based on a specific application's operational needs and not on reporting a single overall performance metric.

Precision and recall measure different operational priorities and should be read together. Precision focuses on the correctness of predicted attacks, while recall (detection rate) focuses on whether attacks are detected. Optimizing recall for safety-critical applications, like healthcare IoMT systems, industrial control networks, or critical infrastructure, is often more crucial, as the impact of an undetected attack can be severe on operations or safety. On the other hand, applications with frequent legitimate traffic variations may prioritize precision to minimize false positive alerts and operator burden. The F1-score is a combination of precision and recall, giving a balanced assessment, and Matthews Correlation Coefficient (MCC) is particularly useful for highly imbalanced datasets, as they take into account all four outcomes of the confusion matrix. Likewise, AUC-ROC provides a threshold-independent assessment of classifier discrimination but should be used in combination with threshold-dependent evaluations because it can overestimate classifier performance in the presence of a very unbalanced class distribution. Table 10 compares the key evaluation metrics for AI-powered IIoT intrusion detection, highlighting the key advantages and drawbacks in a class-imbalanced scenario, as well as the application scenarios for each. This comparison indicates that no single metric is universally suitable and that the choice of metric should be based on the operational goals and deployment needs of the target environment.

**Table 10:** Comparative analysis of performance metrics (Aldhaheri et al., 2023; Swessi and Idoudi, 2022; Sivasankari and Kamalakkannan, 2022).

Evaluation metric	Primary advantage	Principal limitation	Recommended application
Accuracy	Simple overall measure of classification performance	May provide misleading results under severe class imbalance	Balanced datasets; should always be reported together with class-sensitive metrics
Precision	Minimizes false alarms and improves prediction reliability	High precision may still miss a significant number of attacks	Alarm-sensitive environments where reducing false positives is important
Recall (detection rate)	Maximizes attack detection by minimizing false negatives	Higher recall may increase false-positive rates	Safety-critical IIoT, IoMT, and industrial control environments
F1-score	Balances precision and recall into a single metric	Does not explicitly consider true negatives and may mask class-specific weaknesses	General evaluation of imbalanced intrusion detection models
Matthews correlation coefficient (MCC)	Incorporates all confusion matrix outcomes and remains robust under severe class imbalance	Less intuitive to interpret than conventional metrics	Preferred overall metric for highly imbalanced IoT and IIoT intrusion detection datasets
AUC-ROC	Threshold-independent assessment of classifier discrimination	May overestimate performance under severe class imbalance and should be interpreted alongside threshold-dependent metrics	Comparative evaluation of classifiers across different operating thresholds
Inference latency	Measures real-time response capability	Does not evaluate classification quality	Time-critical intrusion detection and edge deployment
Throughput	Measures traffic processing capacity	Does not reflect detection effectiveness	High-bandwidth industrial and enterprise IIoT environments
Memory consumption	Evaluates deployment feasibility on resource-constrained devices	Independent of predictive performance	Edge computing and embedded IoT devices
Computational complexity	Estimates computational resource requirements	Does not directly measure detection accuracy	Model selection for resource-constrained deployments
Energy efficiency	Measures power requirements during inference	Hardware-dependent and not a predictive performance metric	Battery-powered IoT and low-power IIoT devices

As seen in Table 10, no single evaluation metric can completely compare the intrusion detection performance of IIoT. While accuracy is a general measure of classification performance, its value can be misleading when the distribution of classes is highly skewed. Precision and recall represent two other operational objectives and depend on how important false alarms are compared with important attacks. While F1-score offers a balance between these two objectives, MCC is a more complete assessment because it considers all outcomes of the confusion matrix, especially highly imbalanced intrusion detection datasets. Similarly, AUC-ROC is useful for comparing classifiers without specifying a threshold but must be used in conjunction with threshold-dependent measures to obtain a more accurate evaluation of the detection performance in practice.

Beyond predictive performance, practical deployment of AI-driven intrusion detection systems requires evaluation of computational efficiency in addition to classification quality. Models demonstrating excellent predictive performance may remain unsuitable for operational deployment if they exceed the latency, memory, computational, or energy constraints of edge devices. Therefore, future IIoT intrusion detection studies should report deployment-oriented metrics alongside conventional classification metrics. Inference latency determines how rapidly cyber threats can be detected, throughput reflects the capability to process high-volume network traffic, computational complexity and memory consumption determine deployment feasibility on resource-constrained hardware, and energy efficiency is particularly important for battery-powered IoT devices. Consequently, comprehensive evaluation of AI-driven IIoT intrusion detection systems should jointly consider detection effectiveness and deployment efficiency to ensure both high predictive performance and practical applicability in real-world environments.

## 6 Emerging techniques for cyber threat detection

**Figure 8:**
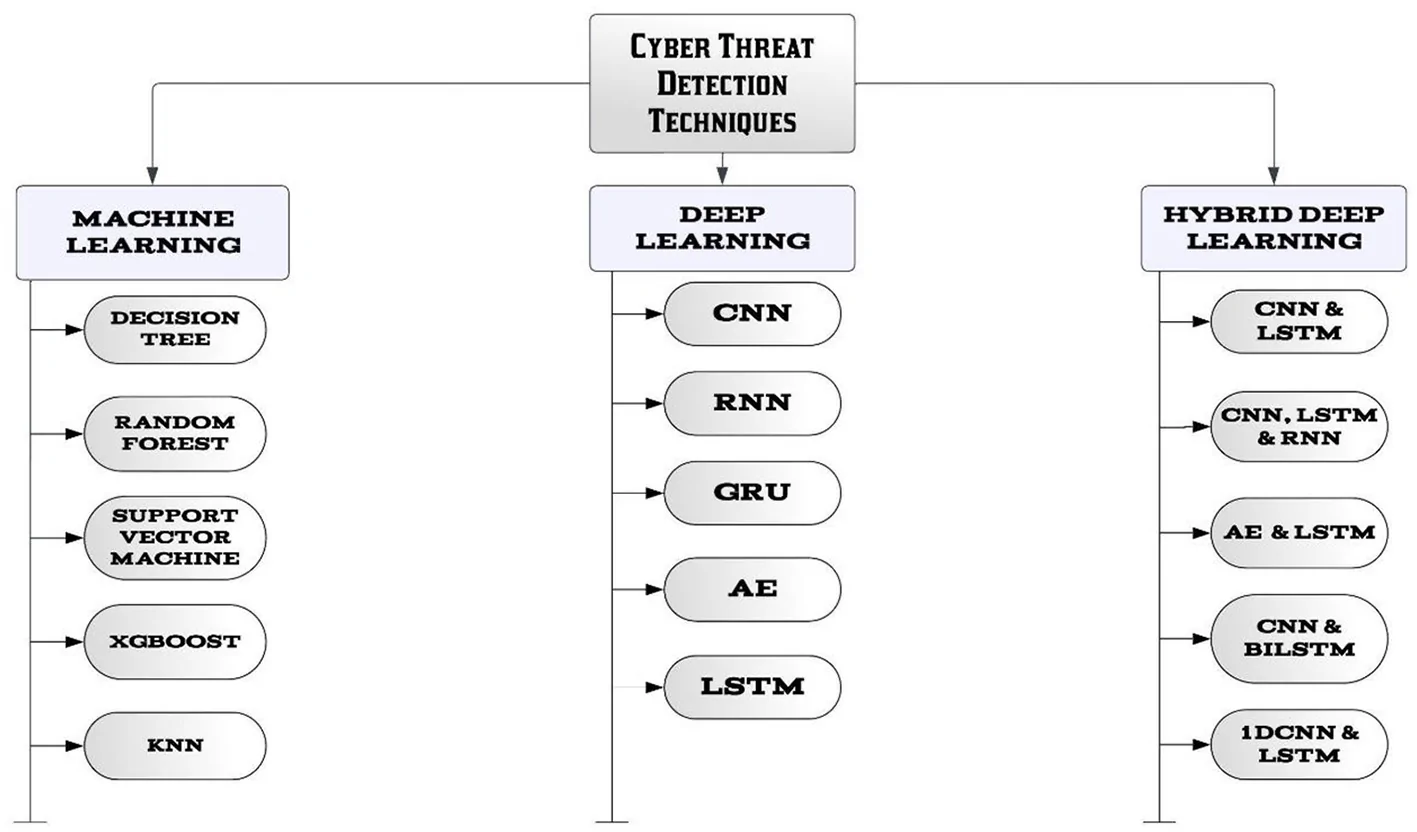
Taxonomy of conventional AI-based cyber threat detection techniques for IIoT security.

Rule-based and signature-based security methods are increasingly insufficient for detecting sophisticated, zero-day, and adaptive cyber threats. The proliferation of IIoT networks yields a steady flow of multidimensional, multiprotocol data from a wide range of devices, and the security community has steadily turned to AI as the key paradigm for adaptive, scalable, and automated threat detection. The evolution has been through three generations: classical machine learning methods, which were explainable and efficient but lacked temporal reasoning and automated features; deep-learning architectures, which regained representational power but became opaque and data-hungry; and hybrid and emerging solutions that are trying to balance accuracy, efficiency, explainability, and privacy across unified detection pipelines. Figure 8 categorizes the fundamental techniques reviewed in this section. Table 11 shows a tabular comparison of representative works across methods and datasets, as well as the strengths and limitations of prior works (Ferrag et al., 2023; Selvarajan et al., 2023).

**Table 11:** Comparative analysis of conventional and emerging AI-based cyber threat detection techniques for IIoT security.

Author	Technique category	Model(s) used	Dataset	Best performance	Key strength	Key limitation
Saba et al. (2022)	Deep learning	CNN	NID, BoT-IoT	Accuracy: 99.51% (NID), 92.85% (BoT-IoT)	Effective on real network traffic	Limited protocol generalizability; no scalability validation
Jain et al. (2022)	Hybrid DL	CNN + Bi-LSTM	NSL-Botnet, UNSW-NB15	Accuracy: 99.4% (NSL-Botnet), 93% (UNSW-NB15)	Spatial + temporal feature capture	Bi-LSTM resource needs unsuitable for lightweight IoT
Elouardi et al. (2024)	Hybrid + LLM	CNN-LSTM, CGAN-BERT, LLMs	X-IIoTID, TON-IoT, CICIoT2023	Survey-based; no unified baseline	First introduction of LLMs (GPT, BERT) into IDS context	No real-time deployment; no experimental baseline
Elnakib et al. (2023)	Deep learning	MLP, CNN, LSTM, CNN+LSTM (EIDM)	CIC-IDS2017	Accuracy: 95%+ (multiclass, 15 classes)	Best multiclass IoT attack classifier in study	Complex; not validated on lightweight or real-time devices
Banaamah and Ahmad (2022)	Comparative DL	CNN, LSTM, GRU	IoT benchmark	CNN: 91.27%; GRU: low FPR; LSTM: best multiclass	Systematic comparison across three DL architectures	High training time; underperforms on complex real-time threats
Awajan (2023)	Deep learning	Deep FCNN	Multi-protocol IoT	Accuracy: 93.74%	Protocol-independent; lightweight architecture	Lower precision; not benchmarked across multiple datasets
Yaras and Dener (2024)	Hybrid DL + Big data	1D-CNN + LSTM (PySpark)	CICIoT2023, TON-IoT	Accuracy: 99.995% (binary), 99.96% (multiclass)	Scalable via Apache Spark; outperforms 10 baselines	No edge/lightweight deployment; no energy analysis
Sajid et al. (2024)	Hybrid ML-DL Feature fusion	XGBoost+LSTM, CNN+LSTM	CIC-IDS2017, UNSW-NB15, NSL-KDD, WSN-DS	High accuracy; low FAR across 4 datasets	Multi-dataset validation; handles unseen attacks	No real-time or resource constraint evaluation
Santhosh Kumar et al. (2023)	Survey + Fuzzy DL	Fuzzy-CNN (proposed)	Survey-based	Proposed only; not experimentally validated	CNN + fuzzy rules for reduced FPR	No implementation or real dataset validation
Jayalaxmi et al. (2022)	Survey + Hybrid framework	ML (SVM, RF, KNN) + DL (CNN, LSTM, AE)	Survey-based	Proposed four-layer hybrid IDPS	Detailed taxonomy; risk-factor mapping strategy	Purely conceptual; no experimental validation
Wahab et al. (2024)	Hybrid DL + SDN	BiLSTM + GRU + SDN	IoT benchmark	Accuracy: 99.93%; reduced FPR	SDN integration for scalability	High resource requirements; complex SDN deployment
Nandanwar and Katarya (2024)	Deep learning	Adaptive CNN-GRU (AttackNet)	IIoT botnet dataset	Detection rate: 99.75%; robust multiclass	High detection; robust to attack type variation	Limited interpretability; no edge resource evaluation
Sharma et al. (2024)	DL + XAI	CNN, DNN + LIME, SHAP	IoT benchmark	High transparency; good detection	Feature-level explainability integrated	Slight accuracy trade-off; added XAI complexity
Musleh et al. (2023)	ML + Transfer features	VGG-16, DenseNet, RF, KNN, SVM	IoT dataset	Accuracy: 98.3% (VGG-16 + stacked)	Multiple feature extractor comparison	No energy/resource analysis; limited generalization
Saheed et al. (2022)	Classical ML	XGBoost, CatBoost, KNN, SVM, NB	UNSW-NB15	Accuracy: 99.9% (PCA + XGBoost)	Compact; PCA-reduced; resource-aware	Single dataset; limited FPR/FNR discussion
Sarhan et al. (2024)	ML + Feature reduction	DFF, CNN, RNN, DT, LR, NB + PCA, AE, LDA	3 IoT benchmarks	Compared 18 model-feature combinations	Cross-dataset generalization analysis	No universal solution; LDA degraded performance
Ferrag et al. (2024)	Foundation model/LLM	Lightweight BERT	IoT/IIoT	Privacy-preserving framework	Resource-aware transformer deployment	Limited real-world deployment validation
García et al. (2025)	Foundation model	TabPFN, TabICL	N-BaIoT and tabular IDS benchmarks	Competitive across multiple datasets	Strong generalization with minimal retraining	Computational complexity
AboulEla and Kashef (2025)	LLM + GNN + XAI	LLM, GNN	Network IDS	Framework proposal	Semantic reasoning and explainability	Limited experimental validation
Li et al. (2024)	Agentic AI	IDS-agent	IoT	Explainable autonomous IDS	Autonomous reasoning	Limited real-world validation; deployment scalability not demonstrated
Ahmed and Alnatheer (2025)	Digital twin	Digital twin IDS	IIoT	Digital twin-enabled IDS	Realistic cyber-physical testing	Limited large-scale evaluation

### 6.1 Machine learning techniques for IIoT security

Classical supervised machine learning approaches, like Support Vector Machines (SVM; Ali et al., 2023; Abiramasundari and Ramaswamy, 2025), Decision Trees (DT; Jones and Omar, 2023; Azam et al., 2023), Random Forests (RF; Chen et al., 2023; Elsersy et al., 2024), k-Nearest Neighbors (KNN; Ali and Al-Sharafi, 2025; Shahbaz et al., 2024), and gradient boosting frameworks like XGBoost (Javed et al., 2024), have served as the baseline paradigm for IDS in IoT environments and remain practical for deployment in resource-constrained environments. The major benefits they offer are well-established: low inference latency, low memory usage, excellent performance on tabular, structured traffic data, and an inherent interpretability that enables operational trust for industrial applications (Bhayo et al., 2023). Random forests have shown consistently excellent generalization on both binary and multiclass intrusion classification problems, built from a large number of decorrelated decision trees that decrease variance without increasing bias (Prity et al., 2024; Routray et al., 2023). The gradient boosting mechanism of the XGBoost algorithm is robust to class imbalance and high-dimensional feature spaces, especially in attack distributions typical of real IIoT deployments (Mahangue et al., 2024; RamKumar et al., 2025). Application of these techniques to the IoT benchmarks has shown false positive rates of less than 1% and accuracy of more than 99%, and use of the Principal Component Analysis (PCA)-based dimensionality reduction has helped to enhance the efficiency without impacting accuracy greatly under controlled conditions.

Nevertheless, classical ML approaches have structural drawbacks that hinder their adoption in the current threat landscape of the IIoT. Manual or semi-automated feature engineering leaves detection quality influenced by the extent to which domain knowledge is captured at design time, which is exacerbated by attack signatures that change or new zero-day attacks (Bhoi et al., 2024; Milić et al., 2023). More fundamentally, traditional ML models assume that each traffic record is independent, ignoring the temporal context and sequential nature of a multi-stage attack campaign, like an advanced persistent threat (APT), lateral movement, or slow-burn reconnaissance. The use of instance-based inference with KNN also restricts scalability in high-volume IIoT streaming applications. All these restrictions combine to spur the drive toward deep learning architectures that can extract features and model temporal sequences autonomously.

### 6.2 Deep learning techniques for IIoT security

As deep learning models have increasingly replaced classic ML in the context of high-performance IoT IDS, they have gained their basis of operation from being able to learn hierarchical feature representations directly from raw or minimally processed data. The three most common families of architectures solve a unique aspect of the IIoT security challenge: convolutional neural networks (CNNs), recurrent architectures (LSTM, GRU, and RNN), and unsupervised architectures (autoencoders).

CNNs (Mittal et al., 2023; Bukhowah et al., 2024; Taşci, 2024) are useful for discovering structured network traffic, structured representations of byte payloads, and system log data with spatially local patterns. These features are translation-invariant and feature-hierarchically abstract, allowing for early detection of subtle traffic anomalies that exceed the accuracy of feature-engineered classifiers based on IoT benchmark datasets, with a reported accuracy of more than 99%. While standard CNNs can effectively process feature maps, they lack “temporal memory” and treat input sequences as static feature maps, reducing their sensitivity to attacks that appear as temporal progressions instead of single signatures.

Recurrent architectures directly tackle this gap. Long Short-Term Memory networks (LSTMs; Inuwa and Das, 2024; Misbha et al., 2024) capture sequential effects over long traffic sequences by learning to retain or forget past information in the system with gating functions, which allow for the detection of emerging attacks and late behavioral clues. Gated Recurrent Units (GRUs; Bala and Behal, 2024; Mir and Trik, 2025) offer similar temporal modeling capabilities but have a simpler architecture that has fewer parameters and can be trained more quickly, making them more suitable for deployment on edge or fog nodes with limited computational resources. Standard RNNs (Raheema, 2025; Hariharan et al., 2025) process data in a similar sequence, but they suffer from vanishing gradients, which makes it difficult to maintain meaningful memory over longer traffic sequences, thus limiting their use to short time series. Comparative studies are presented that show that LSTM is better than standard RNN in binary and multiclass classification of attacks on an IoT system, and that GRU has similar accuracy with fewer false alarms.

Autoencoders (Momand et al., 2023; Harrou et al., 2024) play a special role, as they can be used for unsupervised anomaly detection in environments where labeled attack examples are few, incomplete, or imbalanced, which is typical in operational IIoT deployments. Autoencoders can be trained to reconstruct normal behaviors and detect deviations as potential intrusions by learning the compressed representation of the normal baselines without any explicitly labeled attacks. This property is especially useful in zero-day attack detection, in which there is no previous signature. Variational autoencoders and adversarially trained generative variants enhance these capabilities by creating smoother and more generalizable learned normal behavior manifolds.

Despite the strength of deep learning models, they are structured with cost constraints, which hinders the use of IIoT. The opacity of automated decisions, as there is no human-readable explanation for the results of the classification process, is not just an academic concern but also a regulatory challenge in fields such as healthcare and critical infrastructure, where it is important to audit automated decisions (Aljuhani et al., 2023; Selem et al., 2025). Furthermore, DL models require much more labeled data and computational power to learn than their traditional counterparts, and their weights are fixed throughout the learning process, making them unable to adapt if the IIoT threat landscape evolves. These limitations have led to the development of hybrid and new architectures that combine the representational capabilities of deep learning with the efficiency, interpretability, and adaptability needs of real-world applications of IIoT.

### 6.3 Hybrid frameworks for IIoT security

The latest studies have revealed that hybrid AI models are gaining significance in IIoT cybersecurity. This is because hybrid AI models can address the limitations of machine learning (ML) and deep learning (DL) approaches when used separately. These approaches combine complementary machine learning and deep learning models with explainability and optimization techniques. The latter is more convenient for threats and makes the system more scalable and more transparent. One of the most popular hybrid configurations is CNN-LSTM; it relies on Convolutional Neural Networks to extract the spatial features and the LSTM layer to trace the temporal sequence that simplifies the detection of DoS and DDoS attacks on software-defined networks (Afroj et al., 2024; Ali et al., 2025; Al Shahriar and Dey, 2023; Abdi et al., 2024). This approach has been generalized in other research to CNN-LSTM-RNN hybrids, which provide a layer of temporal representation of intricate multivector incursion in industrial and mobile health settings (Mahdi et al., 2025; Aguru and Erukala, 2024). There are also a few frameworks that use LSTM with autoencoders to do unsupervised anomaly detection and temporal classification at the same time. The approach works with IIoT traffic where the labels are unbalanced or incomplete (Isong et al., 2024; Babaei Goushlavandani et al., 2025). The most recent advanced hybrid systems are starting to employ clustering techniques and blockchain infrastructure to enact decentralized and privacy-safe security procedures at fog computing layers. The application of the Explainable AI (XAI) modules, including LIME and SHAP, to the hybrid models makes operations more transparent, as well. This will assist industrial operators in gaining enhanced knowledge of alerts and confidence in the automated IDS's decisions. Hybrid deep learning pipelines have utilized adaptive intelligence at the edge and transfer learning to improve the generalizability and real-time behavior of pipelines in dynamic industrial operations (Cui et al., 2024; Babar et al., 2025). Hybrid AI models in fields like healthcare and smart grids have proven to more successful in detection rates and also allow real-time decision-making and energy-efficient inference. These architectures, however, have a few issues. They usually require complex training procedures, large datasets, and substantial computing resources, particularly when deployed on edge or heterogeneous infrastructure. Nonetheless, hybrid AI remains a relevant component of IIoT cybersecurity as it provides a balanced solution with flexibility during implementation, an opportunity to understand the ongoing, and a possibility to identify threats (Kandasamy and Roseline, 2025).

### 6.4 Emerging AI paradigms for IIoT cybersecurity

While ML, DL, and hybrid AI models have significantly improved intrusion detection in Industrial Internet of Things (IIoT) environments, recent advances in artificial intelligence have shifted the research focus beyond conventional pattern recognition toward semantic reasoning, autonomous decision-making, and cyber-physical intelligence. Emerging paradigms, including foundation models, large language models (LLMs), agentic AI, digital twins, and multimodal AI, aim to enhance contextual understanding, knowledge transfer, autonomous response, and realistic security validation, thereby representing the next generation of intelligent IIoT cybersecurity frameworks.

#### 6.4.1 Foundation models and large language models (LLMs)

Foundation models have attracted considerable interest because they can be trained on vast amounts of data to produce transferable representations and fine-tuned to handle various cybersecurity tasks without extensive retraining. They also provide better generalization for diverse IIoT environments and different attack patterns than traditional ML models. Ferrag et al. (2024) proved that lightweight, privacy-preserving BERT-based models can be optimized for resource-constrained IoT/IIoT devices, and García et al. (2025) demonstrated that the tabular foundation models (TabPFN and TabICL) can provide competitive intrusion detection performance without requiring intensive task-specific optimization. LLMs augment these capabilities with semantic reasoning, threat intelligence analysis, threat log interpretation, and explainable decision-making. AboulEla and Kashef (2025) also showed how using LLMs in combination with GNNs and Explainable Artificial Intelligence (EAI) enhances contextual intrusion detection and model explainability. However, issues such as computational complexity, domain-specific pretraining, explainability, privacy preservation, and deployment on resource-limited devices remain active research challenges.

#### 6.4.2 Agentic AI and autonomous cyber defense

Agentic AI is an extension of conventional intrusion detection, introducing intelligent agents capable of reasoning, planning, and independently executing security operations. These systems do not just raise alerts; they enable threat investigation, decision-making, and orchestration of responses. Incorporating LLMs with reinforcement learning and retrieval-augmented generation shows promise for creating adaptive and explainable cyber defense systems, as seen in emerging frameworks like IDS-Agent (Li et al., 2024), and L2M-AID (Xu et al., 2025). However, challenges related to trustworthiness, the risk of hallucinations, policy adherence, and the verification of autonomous decisions still need to be addressed before these systems can be safely deployed in safety-critical IIoT environments. Autonomous cyber defense goes beyond intrusion detection and monitoring to incorporate threat detection, reasoning, response, recovery and continuous learning into a closed-loop security model. In contrast to traditional IDSs, which require a manual response once an alert is triggered, autonomous defense systems can coordinate mitigation actions, isolate infected devices, modify security policies, and adjust to new attack patterns over time. The synergies among LLMs, reinforcement learning, and intelligent agents are likely to spur the progress of self-adaptive and resilient IIoT cybersecurity architectures.

#### 6.4.3 Digital twin-based security

The use of digital twins for improved cybersecurity evaluation through realistic cyber-physical simulations is becoming increasingly common. They enable safe attack simulation, anomaly analysis, and intrusion detection validation on virtual replicas of industrial assets and communication networks, without interfering with operational systems. De Benedictis et al. (2023) have proposed a conceptual architecture for anomaly detection using digital twins, and Ahmed and Alnatheer (2025) have demonstrated its use for secure and sustainable IIoT environments. These methods overcome the benchmark realism restrictions and offer a potential base for the creation of predictive and continuously adaptive security mechanisms.

#### 6.4.4 Multimodal AI

Multimodal AI combines multiple data sources such as network traffic, device telemetry, system logs, sensor data, operational context, and other heterogeneous data sources to enhance the understanding of threats across a wider context than just traffic-based intrusion detection. Despite promising outcomes in the wider field of cybersecurity, the use of multimodal learning in the context of IIoT intrusion detection is still quite restricted. This makes multimodal AI a research direction that remains in its early stages and has enormous potential for advancing detection accuracy, explainability, and cross-domain generalization as the capacities of multimodal foundation models continue to improve.

**Figure 9:**
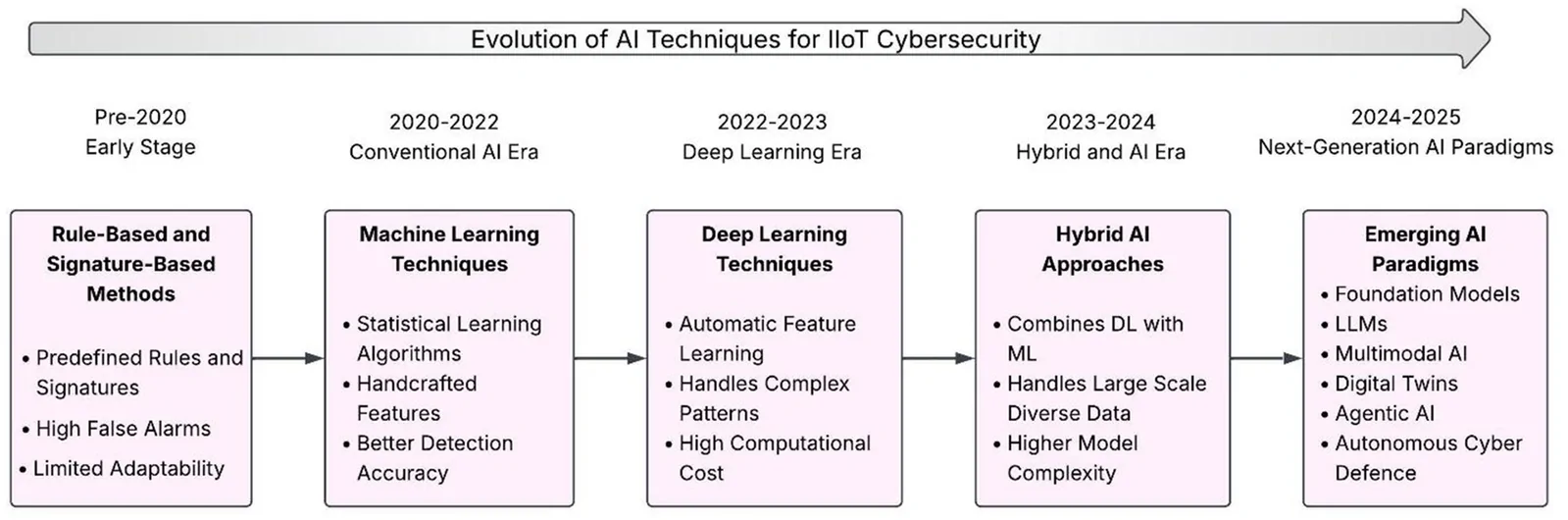
Evolution of AI techniques for IIoT cybersecurity.

These paradigms clearly show the shift of IIoT cybersecurity from traditional supervised classification to intelligent, context-aware, and autonomous defene systems. As shown in Figure 9, AI techniques have evolved from rule-based/traditional machine learning systems to intelligent, context-aware, and autonomous cybersecurity systems in next-generation IIoT environments.

## 7 Open challenges and research gaps in AI-driven IIoT cybersecurity

Despite advances in machine learning, deep learning, hybrid AI, and the new paradigm of intelligence, the shift from benchmark-based intrusion detection applications to real-world operational IIoT cybersecurity remains incomplete. This literature review across this survey shows that many of the reported problems are interrelated and not just research problems. The trustworthiness and feasibility of an AI-based intrusion detection system (IDS) depend on the interplay of four factors: dataset realism, explainability, adversarial robustness, and federated learning, with edge deployment being a critical factor in the trustworthiness and feasibility of an AI-based intrusion detection system (IDS). Future studies could instead focus on integrating several of these AI capabilities to enhance robustness and adaptability, interpretability and privacy preservation, deployment efficiency, and operational resilience.

### 7.1 Data and benchmark realism

Existing studies on intrusion detection are mostly benchmark-based, and most of the frameworks have achieved high accuracy (99% or more) on a few popular datasets like TON-IoT, BoT-IoT, NSL-KDD, and UNSW-NB15. These datasets usually fail to represent the diversity of real IIoT environments, include synthetic attack traffic, or have a well-balanced class distribution. Accordingly, models that achieve good results on a given set of benchmarks often suffer a significant performance drop when tested on independent test sets, on different types of devices, on different network architectures, and in different operational conditions, meaning that the results on a single set of benchmarks are not necessarily meaningful for predicting the performance of models in real-world deployments.

Recent cross-dataset and cross-domain studies strongly support this constraint. Cantone et al. (2024) showed that machine learning-based intrusion detection models that perform near perfectly on the conventional within-dataset evaluation degraded significantly on the cross-dataset evaluation due to distributional differences between benchmark datasets, particularly those involving several combinations of classifiers and datasets that performed almost as randomly as expected by chance. Likewise, de Oliveira et al. (2025) demonstrated that conventional machine-learning models experience substantial performance degradation under cross-dataset evaluation because of distributional differences between benchmark datasets. Their distributed ensemble framework was specifically designed to improve cross-dataset generalization while maintaining scalable intrusion detection performance across heterogeneous network environments. More recently, Kamal and Mashaly (2026) addressed the same limitation by proposing a unified intrusion detection framework that jointly learns representations from multiple heterogeneous benchmark datasets through a shared autoencoder architecture. Their results demonstrate that learning generalized latent representations across heterogeneous datasets improves robustness and generalization by reducing dependence on dataset-specific traffic characteristics. Similarly, Shaikh et al. (2026) conducted a cross-domain IIoT evaluation where models trained on a particular benchmark environment showed a measurable performance drop when evaluated in other distinct operational environments, further highlighting the challenge of benchmark-centric evaluation to ensure a robust deployment.

The problem is exacerbated by concept drift, with the distributions of the IIoT traffic continuously changing because of firmware updates, software updates, topology reconfiguration, new installations, new operation workflows, and adaptive adversarial behavior. As such, an intrusion detection model that has been developed using static historical datasets is increasingly unable to differentiate legitimate and malicious network behavior after deployment. Moreover, continuous evaluation on the same public datasets could also lead to models memorizing the patterns of these specific datasets instead of learning the general intrusion patterns, which might restrict the generalization of the model for use in other heterogeneous industrial networks.

Future IIoT cybersecurity research needs to extend current benchmark-based evaluation and use standardized cross-domain validation methods to evaluate robustness of the deployment in a realistic manner. Instead of taking a random partition of the train-test split from only one benchmark dataset, evaluation protocols ought to be systematically applied on various datasets, differently equipped devices, the temporal evolution of traffic, and various industrial settings. These standardized methods enable a more robust evaluation of generalization and enhance the reproducibility, comparability, and applicability of the systems to real-world IIoT scenarios.

#### 7.1.1 Cross-domain validation methodologies

To bridge the gap between identification and practical methodology, the review suggests a cross-domain standardized validation framework that consists of complementary train-test split methodologies as a set of repeatable experimental designs for future work on intrusion detection in the IIoT. These protocols measure how well-learned representations generalize when tested on new datasets, device types, temporal traffic distributions, and operating contexts, rather than restricting evaluation to idealized benchmark contexts. The standardized versions of these validation methods would also make it easier to compare studies by providing a uniform experimental protocol and reporting of results.

A cross-domain validation framework needs to include five complementary validation protocols. To measure domain generalization, cross-dataset validation should be done by training on one benchmark dataset (e.g., TON-IoT) and testing on another benchmark dataset (e.g., BoT-IoT or CICIoT2023) without retraining. For manufacturer-aware partitioning, it is important to partition the devices belonging to different manufacturers or hardware families to test robustness against device heterogeneity. Temporal partitioning should be used to evaluate resilience to concept drift and changing attack behavior by using chronologically separated traffic. Models should be evaluated in different industrial environments, infrastructures, and network topologies to assess their robustness in real operational scenarios. Finally, the incremental learning evaluation of deployed intrusion detection systems needs to demonstrate the systems' capability to adapt to new attack behaviors learned in the process while still avoiding catastrophic forgetting in long-term operation. The proposed validation protocols should also be reported along with the preprocessing procedures, feature engineering pipelines, hyperparameter settings, random seeds, train-test partition criteria, and evaluation metrics, thereby facilitating comparison and independent replications of future studies on different IIoT benchmark datasets. These validation protocols would be better implemented as standardized reporting protocols rather than experimental settings, thus allowing researchers to distinguish between models that learned transferable intrusion characteristics and those that learned more as a shortcut to artifacts inherent in the benchmark. Table 12 provides an overview of the proposed cross-domain validation framework, recommended train-test split strategies, primary evaluation goals, and reporting criteria for reproducible evaluation of IIoT intrusion detection models. In total, these standardized validation techniques would greatly enhance the trustworthiness, repeatability, and utility of AI-based IIoT cybersecurity studies and offer a more realistic benchmark for the readiness of their deployment for critical industrial infrastructure.

**Table 12:** Reproducible cross-domain validation protocols for IIoT intrusion detection (Cantone et al., 2024; de Oliveira et al., 2025; Kamal and Mashaly, 2026; Shaikh et al., 2026).

Validation protocol	Train-test split strategy	Primary objective	Recommended reporting requirements
Cross-dataset validation	Train on one benchmark dataset (e.g., TON-IoT) and evaluate on an independent benchmark dataset (e.g., BoT-IoT/CICIoT2023) without retraining	Generalization across different data collection environments	Report accuracy, macro-F1, MCC, and source-to-target performance gap
Manufacturer-aware partitioning	Partition training and testing datasets according to device manufacturer or hardware family	Generalization across heterogeneous devices within one deployment	Report per-manufacturer precision, recall, and F1-score together with overall macro-F1
Temporal partitioning	Train using chronologically earlier traffic and evaluate on later traffic from the same deployment	Robustness against concept drift and evolving attack behavior	Report temporal performance decay (accuracy/F1 over time)
Environmental partitioning	Train using traffic collected from one industrial site or topology and evaluate on a different site or topology	Generalization across physical deployment environments	Report source-to-target accuracy/F1 gap and MCC
Continual learning evaluation	Train an initial model and incrementally update it using newly observed traffic	Long-term adaptability while minimizing catastrophic forgetting	Report pre-update and post-update accuracy/F1 together with forgetting metrics

#### 7.1.2 Standardized concept drift benchmarking

Although concept drift has been recognized as a major obstacle to long-term IIoT intrusion detection, systematic longitudinal evaluation remains largely absent from the literature. Most existing studies evaluate models using static train-test partitions, implicitly assuming that network traffic distributions remain unchanged after deployment. In practice, however, IIoT environments continuously evolve because of firmware updates, newly deployed devices, topology reconfiguration, changing operational behavior, and adaptive adversaries, causing the statistical characteristics of network traffic to diverge progressively from those observed during model training. Consequently, intrusion detection performance may deteriorate substantially despite initially achieving excellent benchmark accuracy.

Recent longitudinal evaluation provides quantitative evidence supporting this limitation. Elangovan et al. (2026) evaluated intrusion detection models using four publicly available datasets collected between 2017 and 2024 under forward temporal evaluation without retraining and reported Macro-F1 reductions of approximately 0.20–0.27, together with increased false-positive rates under evolving network environments, demonstrating that conventional within-dataset evaluation substantially overestimates long-term deployment performance. Likewise, Costa et al. (2026) proposed REFINE, a standardized evaluation framework incorporating a Concept Drift Stream Generator (CDSG) and controlled abrupt, gradual, incremental, and recurring drift scenarios, highlighting the need for reproducible drift-aware benchmarking rather than single-point evaluation.

On the basis of these observations, this review introduces a synthetic concept drift benchmark that incorporates three complementary evaluation scenarios: (i) gradual drift (where distributions of traffic features evolve incrementally to simulate, for example, firmware updates, device replacement, and operational changes); (ii) abrupt drift (where new devices, new versions of the firmware, new communication protocols, or new attack variants are introduced at specific points during the evaluation); and (iii) recurring drift (where the distributions of traffic features are periodically observed again to evaluate the continual learning ability and catastrophic forgetting). Future work should assess the intrusion detection systems using longitudinal performance curves (accuracy, macro-F1, or MCC) as well as performance degradation, intrusion detection latency, recovery time after adaptation, adaptation gain, and catastrophic forgetting, rather than reporting a single post-training performance value. Conventional static benchmark testing would be a much less realistic measure of the robustness of long-term deployment of IIoT applications. The proposed benchmark is a systematic evaluation of the robustness of models under complementary drift scenarios, combined with a more traditional static train-test evaluation and with the reporting of temporal degradation and adaptation capability and long-term deployment performance that are kept standardized, as reported in Table 13. With the adoption of these common evaluation procedures, future work to investigate intrusion detection using AI in the IIoT would be more reproducible, comparable, and applicable.

**Table 13:** Proposed standardized longitudinal concept drift benchmark for AI-based IIoT intrusion detection (Elangovan et al., 2026; Costa et al., 2026).

Evaluation scenario	Representative drift simulation	Primary objective	Recommended reporting metrics
Gradual drift	Incremental evolution of traffic distributions caused by firmware updates, device replacement, or operational changes	Measure progressive model degradation	Accuracy, macro-F1, MCC over time, degradation rate (%)
Abrupt drift	Sudden introduction of new device types, firmware versions, communication protocols, or unseen attack variants	Evaluate robustness to rapid distribution shifts	Accuracy loss, drift detection latency, recovery time
Recurring drift	Periodic reappearance of previously observed traffic distributions (e.g., seasonal industrial operations)	Assess continual learning capability and catastrophic forgetting	Recovery accuracy, adaptation gain, forgetting metric

### 7.2 Detection capability against sophisticated threats

While current AI-based intrusion detection systems (IDS) are very effective in detecting statistically distinguishable attacks—DDoS, flooding, brute force, botnet, and more—they are significantly less effective in detecting advanced persistent threats (APTs), insider attacks, SQL injection attacks, the propagation of ransomware, and session hijacking. These attacks are specifically designed to appear like valid industrial communication and would thus need semantic reasoning, context awareness, and behavioral modeling over time, not just statistical outlier detection. This challenge is exacerbated by the presence of adversarial vulnerability since most IDS models are tested in clean benchmark conditions without accounting for adaptive attackers who may be able to generate adversarial network traffic and poison federated model updates.

A critical distinction must be made between two frequently conflated limitations: insufficient representation of stealthy attacks within existing benchmark datasets and architectural limitations of AI models. Evidence indicates that dataset imbalance is the primary bottleneck, whereas architectural limitations become more influential once adequate representative training data are available. Fathima et al. (2026) investigated APT detection under extreme class imbalance (98% benign traffic) using a BERT-based framework incorporating focal loss and hierarchical classification. Although the proposed framework achieved a 95.27% F1-score for binary attack detection, the macro-F1 score decreased to 76.75% for fine-grained multiclass attack categorization under the same architecture, indicating that mitigating class imbalance substantially improves attack detection, while distinguishing among rare attack subtypes remains an architectural challenge.

Recent studies further demonstrate that different AI architectures are better suited to different categories of stealthy threats. Amaru et al. (2025) proposed RAPID, a context-aware self-supervised sequence learning framework that models long-term behavioral dependencies within system provenance data, demonstrating strong potential for multi-stage APT detection while reducing false positives. Similarly, foundation models and large language models provide semantic reasoning capabilities that are well-suited for detecting SQL injection and other context-dependent attacks, graph neural networks effectively model interactions among users, devices, and network entities for insider threat detection, whereas multimodal and continual learning frameworks offer promising solutions for ransomware detection by integrating heterogeneous information sources and continuously adapting to evolving attack behaviors. Table 14 summarizes representative stealthy attack categories, their primary detection challenges, promising AI architectures, and recommended evaluation metrics.

**Table 14:** AI-based architectures and recommended metrics for IIoT threat detection (Fathima et al., 2026; Amaru et al., 2025).

Sophisticated threat	Primary detection challenge	Promising AI architecture	Recommended evaluation metrics
Advanced persistent threat (APT)	Long-term multi-stage attack behavior	Context-aware transformer-based sequence models, Self-supervised learning	Macro-F1, MCC, per-stage recall
SQL injection	Semantic similarity to legitimate communication	Foundation models, large language models	Macro-F1, MCC, precision-recall AUC
Ransomware	Rare and continuously evolving attack behavior	Multimodal learning, continual learning	Macro-F1, MCC, balanced accuracy
Insider threat	Context-dependent behavioral anomalies	Graph neural networks, behavior modeling	Macro-F1, per-class recall, MCC
Session hijacking	Sequential session correlation and behavioral dependency	Attention-based sequential models	Macro-F1, MCC, balanced accuracy

Because stealthy attacks typically represent minority classes, overall accuracy should not be considered the primary evaluation metric. Future IIoT intrusion detection studies should instead prioritize Macro-F1, Matthews Correlation Coefficient (MCC), per-class Recall, Balanced Accuracy, and Precision-Recall AUC, which provide a more reliable assessment of detection capability under severe class imbalance. Collectively, these methodological recommendations establish a more rigorous evaluation framework for detecting sophisticated IIoT cyber threats under realistic deployment conditions.

#### 7.2.1 Standardized adversarial robustness evaluation

While adversarial machine learning has emerged as an active research field, only a small number of studies on IIoT intrusion detection rigorously assess robustness against adversarial attacks. Most published IDS models do not report performance when network traffic is maliciously perturbed; this lack of reporting has not been explored extensively. Adversarial perturbations generated with the Fast Gradient Sign Method (FGSM), Projected Gradient Descent (PGD), and Carlini–Wagner (C&W) attacks have been shown to substantially degrade intrusion detection performance in recent studies, in line with the claims of other recent studies, which indicates that high benchmark accuracy does not ensure secure deployment. For instance, Awad et al. (2025) noted that a DNN-based IDS with an accuracy of 98.11% on clean traffic dropped to 54%, 45%, and 36% for FGSM, Basic Iterative Method (BIM), and C&W attacks, respectively. In a similar fashion, Roshan et al. (2023) observed that adversarial training achieved robust detection accuracy of 98.70% (FGSM), 98.68% (PGD), and 98.47% (JSMA), while the accuracy against C&W attacks was still significantly low at 71.56%, suggesting that optimization-based attacks remain the biggest challenge. Additionally, Wang and Yan (2025) introduced an Auxiliary Adversarial Training Wasserstein GAN (AuxAtWGAN) system and systematically compared the adversarial attacks of FGSM, PGD, and C&W, showing that adversarial training could significantly boost robustness while keeping the detection performance on clean traffic competitive. The overall results suggest that adversarial robustness should be taken into account as a benchmark metric along with traditional detection metrics for building reliable and secure IIoT IDS. A summary of the representative adversarial attack benchmarks and the recommended defense strategies is presented in Table 15.

**Table 15:** Standardized adversarial robustness benchmarks for AI-based IIoT IDS (Awad et al., 2025; Roshan et al., 2023; Wang and Yan, 2025).

Adversarial attack	Representative performance degradation	Recommended defense	Representative effectiveness
FGSM	Accuracy reduced from 98.11% to 54% in a representative DNN-based IDS	Adversarial training	Accuracy recovered to 98.70% after adversarial training
PGD	Produces greater performance degradation than FGSM under iterative perturbation settings	Auxiliary adversarial training (AuxAtWGAN)	Accuracy recovered to 98.68% after adversarial training
Carlini–Wagner (C&W)	Accuracy reduced from 98.11% to 36%, representing the strongest evaluated attack	Ensemble defense + Adversarial training	Recovery remained limited (71.56%), indicating higher attack resilience requirements
Recommended benchmark	Evaluate every IDS using FGSM, PGD, and C&W under standardized perturbation settings (e.g., identical ε, iteration count, and attack configuration).	Standardized robustness evaluation	Report clean accuracy, robust accuracy, attack success rate (ASR), macro-F1, MCC, perturbation magnitude (ε), and inference latency

Adversarial robustness should be used as a standard benchmarking requirement for future IIoT intrusion detection studies and not as an optional experiment to prove the study's adversarial robustness. Along with traditional detection measures, future detection measures should provide consistent reporting of clean accuracy, robust accuracy, attack success rate (ASR), robustness degradation, macro-F1, MCC, perturbation magnitude (ε), and inference latency, along with standardized attack configurations. A common basis of adversarial robustness assessments, such as the one proposed here, would allow independent evaluation of defenses and speed up the progress of AI-driven intrusion detection systems in safety-critical IIoT settings.

### 7.3 Deployment, scalability, and efficiency

While high detection accuracy is beneficial, it is not enough to ensure successful deployment in resource-limited IIoT applications. Modern architectures such as transformers, graph neural networks, and deep hybrid models are computationally and memory-intensive and consume a lot of energy, which puts them out of reach of edge devices for early threat detection. Moreover, federated learning is a promising training paradigm for privacy-preserving training; however, it is typically assessed with unrealistic independently and identically distributed (IID) scenarios, where the devices' properties, communication conditions, and operational behaviors do not reflect the real-world characteristics of industrial deployments. The lack of integration of DP, secure aggregation, and scalable federated optimization further limits practical deployment. Future studies, therefore, explore edge-native lightweight AI architectures, TinyML, federated optimization under non-IID data, energy-aware model design, and privacy-preserving distributed learning to develop scalable and efficient cybersecurity in the IIoT.

#### 7.3.1 Standardized energy metrics and energy-efficiency evaluation framework

While computational complexity and inference latency are often mentioned in AI-based studies of IIoT IDS, there is a lack of a standardized energy-efficiency evaluation framework. Most current research focuses on the detection accuracy of continuous intrusion monitoring without accounting for the energy consumption of resource-constrained IIoT devices. This makes it difficult to objectively compare the performance of different AI architectures and to make deployment decisions. To overcome this, the current review suggests a standardized energy evaluation framework, which is made up of three complementary metrics.

- Joules per Detection (J/detection) measures the average energy required to classify a single network sample by dividing the total inference energy by the number of processed samples.
- Latency per Watt (ms/W) jointly evaluates inference responsiveness and power efficiency by normalizing inference latency with average power consumption, where lower values indicate superior energy-performance trade-offs.
- Battery Runtime on Reference Edge Hardware estimates the continuous operating lifetime of an IDS deployed on standardized platforms, such as Raspberry Pi 4 or Raspberry Pi 5, by relating battery capacity (Wh) to measured average power consumption (W), thereby providing a practical indicator of deployment sustainability.

Energy profiling should be conducted under standardized conditions and with the same data sets, preprocessing pipelines, train-test sets, operating systems, and edge hardware as they are in future studies to enable reproducible and fair comparisons across studies. Energy measurement should be done by external energy measuring instruments, if possible, to ensure the accuracy and repeatability of the measurement, in contrast to software energy measurement. It is suggested that during inference, the average power consumption, latency, CPU utilization, memory footprint, throughput, and energy consumption be measured following a consistent profiling methodology that enables the calculation of the proposed standardized metrics.

There has been a recent trend of including energy-aware evaluation in edge-deployed IIoT intruder detection systems. Diab et al. (2025) showed that the hardware-aware optimization significantly decreases the computational and energy demands of deep learning models running on the Raspberry Pi 3B+, while the light algorithms they used, such as LightGBM and XGBoost, produced significantly lower inference energy than traditional CNN architectures. Umar et al. (2025) proposed an energy-efficient deep learning intrusion detection framework based on knowledge distillation and quantization for resource-constrained edge environments. Their DNN-KDQ model substantially reduced the model size while maintaining high detection accuracy and achieving ultra-low inference latency, demonstrating the feasibility of deploying lightweight AI-based intrusion detection on edge devices through model compression and energy-aware optimization. Likewise, Alharbi et al. (2024) have tested the feasibility of implementing a lightweight CNN–LSTM intrusion detection system on the fog nodes using a Raspberry Pi platform for real-time continuous monitoring with reasonable power consumption. These studies provide evidence of the increasing trend for IDS to deploy energy conservation techniques and also show that there is no uniformity between different literature sources in terms of the energy evaluation techniques used. These are typical energy characteristics summarized in Table 16.

**Table 16:** Energy characteristics for edge-deployed AI-based intrusion detection systems (Diab et al., 2025; Umar et al., 2025; Alharbi et al., 2024).

References	Architecture(s) evaluated	Edge hardware	Reported energy characteristics	Key findings
Diab et al. (2025)	LightGBM, XGBoost, hardware-aware 1D-CNN, conventional CNN	Raspberry Pi 3B+	Tree-based models achieved inference latency below 30 ms with energy consumption below 7 mJ/prediction, whereas conventional CNNs required >82 mJ/prediction. Hardware-aware optimization reduced the 1D-CNN footprint to 190 KB with 840 K FLOPs.	Hardware-aware optimization significantly improved deep learning energy efficiency, while lightweight machine learning models remained the most suitable for resource-constrained edge deployment.
Umar et al. (2025)	DNN-KDQ (knowledge distillation quantization)	Raspberry Pi 4	Model size reduced from 196.77 to 20.18 KB with an inference time of 0.07 ms per sample, enabling lightweight real-time IDS deployment on resource-constrained edge devices.	Demonstrated that model compression through knowledge distillation and quantization can substantially improve deployment efficiency while maintaining high intrusion detection accuracy for edge-based IDS.
Alharbi et al. (2024)	Lightweight CNN–LSTM IDS	Raspberry Pi fog node	Maximum power consumption of 6.12 W during continuous real-time inference.	Demonstrated that optimized hybrid deep learning architectures can achieve real-time intrusion detection on Raspberry Pi-based fog nodes with practical power requirements.

The reported measurements originate from independent studies employing different datasets, hardware platforms, software implementations, and experimental methodologies. Therefore, the reported values represent indicative energy characteristics rather than direct quantitative comparisons among AI architectures.

As summarized in Table 16, the studies indicated that energy-aware IIoT intrusion detection is becoming more of a focus, although there is still a lack of standardization in the evaluation methodologies, which makes cross-study comparison more difficult. Conventional performance indicators such as accuracy, precision, recall, F1 score, and AUC should therefore be supplemented by future studies reporting the Joules per Detection, Latency per Watt, Battery Runtime, average power consumption, CPU utilization, memory footprint, and throughput using a fixed edge hardware setup. If these common metrics can be adopted, then energy-performance trade-offs can be reproducibly evaluated, energy-efficient IDS systems can be fairly compared across different AI architectures, and energy-efficient IDS systems can be deployed at a greater rate within resource-constrained IIoT environments.

#### 7.3.2 Non-IID federated learning and privacy-preserving optimization

Although federated learning (FL) has emerged as a promising privacy-preserving paradigm for distributed IIoT intrusion detection, most existing studies continue to evaluate FL under independently and identically distributed (IID) assumptions that rarely reflect practical industrial deployments. Recent IIoT intrusion detection studies demonstrate that statistical heterogeneity severely affects model performance, particularly for minority attack classes. Under compromised-device conditions using conventional FedAvg, benign traffic classification remained high (94.44%), whereas attack-class accuracy decreased to 23.22%, indicating that aggregate accuracy can conceal substantial degradation in security-critical attack detection. Heterogeneity-aware aggregation strategies have reported up to 71.07% improvement in minority attack-class accuracy over FedAvg/FedProx with only 4.7% (5.03 s) additional communication latency per training round, while personalized federated learning combined with knowledge distillation achieved F1-scores exceeding 98% for previously unseen attack classes compared with approximately 50% for conventional FedAvg and FedProx.

Recent studies increasingly evaluate FL using Dirichlet-distributed client partitions, where the concentration parameter α controls statistical heterogeneity (α = 0.1 indicates severe non-IID conditions, whereas α = 1.0 approaches IID data). Furthermore, although federated learning protects raw data privacy, exchanged model updates remain vulnerable to gradient inversion and membership inference attacks. Consequently, recent FL frameworks increasingly integrate Differential Privacy (DP), where the privacy guarantee is quantified by the privacy budget ε, requiring careful balance between privacy protection and detection performance. The representative characteristics of commonly adopted federated aggregation strategies are summarized in Table 17. Future IIoT federated intrusion detection studies should systematically report Dirichlet heterogeneity (α = 0.1, 0.3, 0.5, 1.0), Differential Privacy budget (ε), attack-class performance, communication overhead, convergence rounds, and Byzantine robustness alongside conventional accuracy and F1-score. Such standardized reporting would enable objective comparison of privacy-preserving federated intrusion detection systems under realistic heterogeneous industrial environments.

**Table 17:** Comparison of representative federated aggregation strategies for non-IID IIoT intrusion detection.

Aggregation strategy	Non-IID handling mechanism	Performance under heterogeneity	Privacy/robustness characteristics
FedAvg	Simple parameter averaging	Baseline; attack-class accuracy reduced to 23.22% under severe heterogeneity	High communication efficiency; limited robustness to non-IID data
FedProx	Proximal regularization to reduce client drift	More stable than FedAvg but still degrades under highly skewed client distributions	Improved convergence under heterogeneous devices
Heterogeneity-aware aggregation	Minority-aware/client-aware aggregation	Up to 71.07% improvement in minority attack-class accuracy over FedAvg/FedProx with 4.7% (5.03 s) additional latency	Improved robustness to statistical heterogeneity
Personalized FL + Knowledge distillation	Client-specific personalization with shared knowledge	>98% F1 for unseen attack classes compared with ≈50% for FedAvg/FedProx	Strong adaptability under heterogeneous local data
Krum/Multi-Krum	Byzantine-resilient aggregation	Moderate robustness to non-IID data	High resilience against malicious client updates
Trimmed mean/median	Robust aggregation excluding abnormal updates	Stable under poisoning attacks	Improved resistance to adversarial model updates

The comparison is synthesized from the representative federated learning studies reviewed in this section and summarizes the relative characteristics of commonly adopted aggregation strategies under heterogeneous IIoT environments. Reported performance values are representative of the cited literature and are intended for comparative analysis rather than direct quantitative comparison.

### 7.4 Trust, explainability, and standardization

The widespread deployment of AI-based IDS in the critical infrastructure of the Internet of Things (IIoT) relies not only on predictive performance but also on transparency, robustness, and reproducibility. Most of the best-performing IDS models are black-box systems that are difficult for security analysts, system operators, and regulatory bodies to interpret, especially in safety-critical areas subject to standards like IEC 62443, GDPR, and the NIST Cybersecurity Framework. While they can yield valuable *post-hoc* insights such as explainability techniques like SHAP, LIME, and attention visualization, they can also add to the computational burden and may provide only approximate explanations. At the same time, the absence of standard datasets, pre-processing procedures, train-test protocols, adversarial evaluation methods, reporting for different tiers of deployments, and performance metrics hinders meaningful comparisons across studies and satisfactory reproducibility. Future research should focus on creating intrinsically interpretable AI models, trustworthy AI frameworks, standard evaluation protocols, and community-wide benchmarking practices that provide greater transparency, reproducibility, and regulatory compliance in IIoT cybersecurity.

The identified research gaps should be considered as a comprehensive problem and not as a succession of technical gaps. The future IIoT intrusion detection systems will need to integrate complementary AI paradigms that address the challenges of generalization, detection of sophisticated threats, deployment efficiency, preserving privacy and security, explainability, and trustworthy decision-making, as summarized in Table 18. Future research should go beyond fine-tuning individual detection methods and aim to create comprehensive AI ecosystems that combine reliable intelligence, continuous learning, semantic understanding, privacy protection, effective edge deployment, and standardized assessment. These comprehensive solutions will play a critical role in supporting robust, scalable, and deployable IIoT cybersecurity solutions that can help protect an ever-more complex industrial landscape.

**Table 18:** Unified research roadmap linking consolidated research challenges with AI-based solution directions.

Consolidated challenge	Key research issues	AI-based solution directions	Priority research focus	Expected outcome
Data and benchmark realism	Dataset bias, benchmark saturation, concept drift	Cross-dataset validation, standardized cross-domain validation, manufacturer-aware train-test partitioning, temporal validation, continual learning	Robust generalization across heterogeneous devices, industrial environments, and evolving traffic distributions	Adaptive, reproducible, and deployment-ready intrusion detection
Detection capability against sophisticated threats	Stealthy attacks, adversarial attacks	Foundation models, LLMs, multimodal AI	Semantic threat understanding	Robust detection of advanced cyber threats
Deployment, scalability, and efficiency	Edge constraints, non-IID federated learning	TinyML, edge AI, federated learning	Practical large-scale deployment	Energy-efficient privacy-preserving IDS
Trust, explainability, and standardization	Black-box AI, inconsistent evaluation	Explainable AI, trustworthy AI	Transparent and reproducible AI	Regulation-compliant cybersecurity

#### 7.4.1 Explainability-performance trade-offs and regulatory alignment

Explainable AI (XAI) is an emerging research area for AI-based intrusion detection systems, and it is the focus of most existing research, but it is evaluated qualitatively without taking into account the computational complexity or deployment challenges of explainability. *Post-hoc* explanation techniques are always accompanied by extra inference delay, as explanations are produced after model prediction, and there is generally a practical compromise between detection accuracy, explanation fidelity, computational efficiency, and real-time deployment. Thus, explainability should be considered a system characteristic that can be measured, not merely a visualization element.

SHAP is among the most popular *post-hoc* explainability methods and typically yields high-fidelity representations of feature attribution, but it has high computational costs, especially for deep neural networks and complex ensemble models, due to repeated Shapley-value estimation. LIME usually provides explanations faster by using a simpler model that looks at a small part of the data, but it can be less consistent when run multiple times because it relies on random changes in the data. Compared with intrinsically interpretable models, such as decision trees and rule-based classifiers, which offer interpretable decision-making without needing *post-hoc* explanation algorithms, attention visualization adds relatively low additional computational overhead during model inference. Beyond model interpretation, explainability has been shown to enhance deployment efficiency in recent studies. For instance, SHAP-guided feature selection and knowledge distillation have been applied to build lightweight student models that significantly limit latency for inference and memory consumption in edge deployment scenarios with moderate drops in detection accuracy, showing how explainability can be beneficial in designing resource-efficient IDS.

In addition to the computational aspect, explainability has emerged as a key feature for the deployment of trustworthy AI that is trustworthy. Both the General Data Protection Regulation (GDPR) article 22 on meaningful information about automated decision-making when it has a significant effect on individuals and IEC 62443, which focuses on traceability, accountability, and auditable security decisions in industrial automation and control systems, highlight the importance of providing meaningful information. Therefore, future IIoT intrusion detection systems must be optimized to provide a balance of detection accuracy, explanation fidelity, computational efficiency, and regulatory compliance to ensure that they can be deployed safely and reliably in safety-critical industrial infrastructure. Table 19 presents summary statistics of the representative explainability approaches used.

**Table 19:** Comparison of representative explainability approaches for AI-based IIoT intrusion detection.

AI model/ explainability approach	Relative explanation overhead	Explanation fidelity	Accuracy–explainability trade-off	GDPR Art. 22 alignment	IEC 62443 alignment
Decision tree	Very low	High	Complete interpretability with moderate predictive performance	High	High
Rule-based IDS	Very low	High	Fully transparent but limited adaptability to evolving attacks	High	High
Random forest + TreeSHAP	Low	High	Maintains high detection performance with efficient feature attribution	High	High
XGBoost + TreeSHAP	Low	High	High predictive performance with moderate explanation cost	High	High
LightGBM + TreeSHAP	Low	High	Well-suited for real-time explainable edge deployment	High	High
CNN + SHAP	High	High	Superior detection accuracy at the expense of increased explanation latency	Moderate	High
CNN–LSTM + SHAP	High	High	Improved temporal learning with higher computational overhead	Moderate	High
Transformer + attention visualization	Low–moderate	Moderate–high	Efficient intrinsic visualization with limited causal interpretability	Moderate	High
Graph neural network + GNNExplainer	High	High	Rich structural explanations with substantial computational cost	Moderate	High
Black-box models + LIME	Moderate	Moderate	Faster explanations than SHAP but lower stability and reproducibility	Moderate	Moderate

The qualitative ratings are synthesized from the representative studies reviewed in this section and are intended for relative comparison. Explanation Overhead reflects the additional computational cost of generating explanations, whereas Explanation Fidelity indicates how faithfully explanations represent model decisions. GDPR Article 22 and IEC 62443 Alignment indicate the relative suitability of each approach for supporting explainable and trustworthy AI in industrial cybersecurity.

Table 19 provides a comparison, showing that no single explainability approach can maximize predictive performance, computational efficiency, explanation fidelity, and regulatory compliance at the same time. Significantly more computational resources are needed to produce high-fidelity explanations with deep learning architectures, while lightweight machine learning models together with TreeSHAP currently offer one of the most favorable trade-offs for resource-constrained IIoT deployments. Finally, recent explainability-guided model compression methods suggest that XAI may optimize computational efficiency by eliminating irrelevant features and transferring knowledge to or from the target model. Hence, to make it easier to compare trustworthy AI systems in safety-critical industrial applications, future IIoT intrusion detection research should build on conventional security and safety metrics by reporting the same set of explanation latency, computational overhead, explanation fidelity, stability, and regulatory compliance.

## 8 Discussion and future research directions

This review demonstrates how AI has revolutionized IIoT cybersecurity, moving beyond the traditional signature-based intrusion detection to intelligent, adaptive, and data-driven security models. From machine learning laying the groundwork for automated threat detection to deep learning, hybrid architectures, transformer models, graph neural networks, federated learning, reinforcement learning, and explainable AI, the technical evolution of threat detection has evolved to provide more accurate detection, contextual understanding, and collaborative intelligence. Recent trends like foundation models, large language models (LLMs), multimodal AI, digital twins, and agentic AI are expected to shape the future of IIoT cybersecurity, going beyond traditional anomaly detection methods to facilitate semantic reasoning, autonomous decision-making, and intelligent cyber defense.

Instead of simply fine-tuning individual detection methods on benchmark datasets, future cybersecurity research in the field of IIoT should be dedicated to creating an integrated AI ecosystem of trustworthy intelligence, distributed learning, continuous adaptation, contextual reasoning, and standardized evaluation. The findings of this review are summarized in Table 20, which classifies future research directions by priority as determined by their anticipated implementation timeframe and strategic value.

**Table 20:** Prioritized research roadmap for the evolution of AI-driven IIoT cybersecurity.

Time horizon	Research priorities	Representative AI technologies	Expected impact
Short-term (1–2 years)	Cross-dataset validation, longitudinal benchmarking, standardized evaluation protocols, intrinsically interpretable AI, trustworthy AI	Explainable AI (XAI), continual learning, standardized benchmarking	Improved reproducibility, trustworthy decision-making, and regulatory compliance
Medium-term (2–4 years)	Edge-native lightweight architectures, scalable federated learning, semantic threat understanding, multimodal threat analysis	TinyML, edge AI, federated learning, differential privacy, LLMs, graph neural networks	Practical, scalable, privacy-preserving, and context-aware IIoT cybersecurity
Long-term (4+ years)	Digital twin-enabled cybersecurity, foundation models, agentic AI, autonomous incident response, self-healing cybersecurity ecosystems	Foundation models, digital twins, agentic AI, reinforcement learning	Fully autonomous, adaptive, resilient, and self-managing industrial cybersecurity

In the proposed roadmap, the focus is clearly shifting from evaluation reliability and deployment readiness toward intelligent, autonomous, and self-adaptive IIoT cybersecurity. Further research should focus on realistic benchmarks, standardized assessment, and trustworthy AI to ensure robust groundwork for implementation. Medium-term, scalable edge intelligence, semantic threat understanding, and privacy-preserving distributed learning for efficient operations in diverse industrial settings need to be addressed. In the longer term, adoption of foundation models, multimodal intelligence, digital twins, agentic AI, and continual learning will enable the creation of autonomous and self-healing cybersecurity ecosystems that can proactively safeguard next-generation IIoT infrastructures. To realize this vision, it will take a joint effort in the development of interoperable AI frameworks, in adopting realistic validation processes, and in defining standardized deployment protocols to move beyond the laboratory and into real-world IIoT deployments.

## 9 Conclusion

This review brings together a wide range of recent works on AI-based IIoT cybersecurity under a unified analytical framework to provide a comprehensive and systematic assessment of the progress in AI-based intrusion detection methodologies, publicly available benchmarks, industrial cyber threats, evaluation metrics, emerging AI paradigms, and open research challenges. In addition to summarizing the literature, this review provides a structured taxonomy of AI-intrusion detection techniques, expands comparative analyses with deployment-oriented selection and evaluation frameworks of datasets, and brings together the fragmented research challenges and future AI technologies into a prioritized roadmap to match practical needs for IIoT cybersecurity.

The analysis demonstrates that in the recent past, the use of machine learning, deep learning, transformer architectures, graph neural networks, federated learning, reinforcement learning, and explainable AI has greatly enhanced the capacity of intrusion detection systems. However, one of the main outcomes of this review is that high benchmark performance is no longer sufficient to evaluate operational cybersecurity performance. To ensure reliable operation in the diverse industrial domain, future AI-based intrusion detection systems need to integrate strong cross-domain generalization, explainability, adversarial resilience, privacy preservation, continual adaptation, computational efficiency, and deployment readiness.

The findings of this review help researchers, developers, and industrial practitioners by outlining what capabilities are needed for next-generation IIoT IDS. The deployment-oriented dataset selection framework, the extended evaluation criteria, and the prioritized research roadmap included in this review will serve as valuable guidance for selecting appropriate AI methodologies, assessing model performance beyond the traditional accuracy metrics, and aligning future research directions with realistic industrial cybersecurity needs.

In general, the future of IIoT cybersecurity will be defined by the combination of complementary AI paradigms, not by the optimization of separate learning algorithms. The integration of explainable AI, continual learning, federated intelligence, multimodal reasoning, foundation models, digital twins, and agentic AI presents a compelling pathway toward intelligent, adaptive, trustworthy, and self-managing cybersecurity ecosystems that can safeguard next-generation industrial infrastructure against growing cyber threats.
